# Pangenomic analyses of antibiotic-resistant *Campylobacter jejuni* reveal unique lineage distributions and epidemiological associations

**DOI:** 10.1099/mgen.0.001073

**Published:** 2023-08-01

**Authors:** Jose A. Rodrigues, Heather M. Blankenship, Wonhee Cha, Sanjana Mukherjee, Rebekah E. Sloup, James T. Rudrik, Marty Soehnlen, Shannon D. Manning

**Affiliations:** ^1^​ Department of Microbiology and Molecular Genetics, Michigan State University, East Lansing, MI, USA; ^2^​ Michigan Department of Health and Human Services, Bureau of Laboratories, Lansing, Michigan, USA; ^†^​ Present address: National Veterinary Institute, Uppsala, Sweden; ^‡^​ Present address: Center for Global Health Science and Security, Georgetown University, Washington, USA

**Keywords:** antibiotic resistance, *Campylobacter jejuni*, epidemiology, genomics, pangenome

## Abstract

Application of whole-genome sequencing (WGS) to characterize foodborne pathogens has advanced our understanding of circulating genotypes and evolutionary relationships. Herein, we used WGS to investigate the genomic epidemiology of *Campylobacter jejuni *, a leading cause of foodborne disease. Among the 214 strains recovered from patients with gastroenteritis in Michigan, USA, 85 multilocus sequence types (STs) were represented and 135 (63.1 %) were phenotypically resistant to at least one antibiotic. Horizontally acquired antibiotic resistance genes were detected in 128 (59.8 %) strains and the genotypic resistance profiles were mostly consistent with the phenotypes. Core-gene phylogenetic reconstruction identified three sequence clusters that varied in frequency, while a neighbour-net tree detected significant recombination among the genotypes (pairwise homoplasy index *P*<0.01). Epidemiological analyses revealed that travel was a significant contributor to pangenomic and ST diversity of *C. jejuni *, while some lineages were unique to rural counties and more commonly possessed clinically important resistance determinants. Variation was also observed in the frequency of lineages over the 4 year period with chicken and cattle specialists predominating. Altogether, these findings highlight the importance of geographically specific factors, recombination and horizontal gene transfer in shaping the population structure of *C. jejuni *. They also illustrate the usefulness of WGS data for predicting antibiotic susceptibilities and surveillance, which are important for guiding treatment and prevention strategies.

## Data Summary

Sequencing reads used in the analysis were deposited in the National Center for Biotechnology Information (NCBI) within BioProjects PRJNA305291, PRJNA368990 and PRJNA951423. Strain accession numbers and metadata are shown in Table S1, available in the online version of this article. Bioinformatic scripts are available at: https://github.com/RodriguesJA/Pangenomic-diveristy-of-C.-jejuni, while the phylogeographical data are accessible through Microreact. All authors confirm support of data, codes and protocols noted within the article.

**Impact Statement**

*
Campylobacter jejuni* is a Gram-negative pathogen and the leading cause of gastroenteritis worldwide. Use of whole-genome sequencing and pangenomic analyses enabled a comprehensive assessment of the *C. jejuni* strain types (lineages) linked to infections in patients from Michigan, USA. Risk factors for specific lineages and infection sources were identified with some lineages being associated with residence location, recent travel history, cattle or chicken sources, and carriage of clinically important antibiotic resistance genes. Identifying these high-risk lineages in circulation in different geographical locations is critical for guiding the development of more targeted prevention strategies.

## Introduction

*Campylobacter* spp. are Gram-negative bacterial pathogens and leading causes of gastroenteritis, or campylobacteriosis, worldwide [1]. According to the Centers for Disease Control and Prevention (CDC), which monitors foodborne pathogens through the Foodborne Disease Surveillance Network (FoodNet), *Campylobacter* causes 1.5 million human infections in the USA each year [2], with most being caused by *C. jejuni *. Campylobacteriosis is often self-limiting with patients reporting symptoms of diarrhoea, cramping, abdominal pain and vomiting that typically last between 7 and 10 days [1]. International travel was found to be the most important risk factor for *Campylobacter* infections in a prior meta-analysis along with consuming undercooked chicken and acquisition from the environment or via direct contact with farm animals [3].

Fluoroquinolones and macrolides are first-line antibiotics used to treat campylobacteriosis; however, resistance to both has increased in frequency, thereby limiting treatment options [2]. Consequently, the CDC has classified drug-resistant *Campylobacter* spp. as a ‘serious’ public health threat contributing to 448 400 infections and 70 deaths each year [2]. To monitor trends in resistance across the FoodNet sites, the National Antimicrobial Resistance Monitoring System (NARMS) was created in 1996 [4]. However, only data from the ten FoodNet sites are monitored, which represents 5–10 % of the US population and does not include Michigan [5]. Our prior studies of *C. jejuni* [6, 7] and non-O157 Shiga toxin-producing *Escherichia coli* [8] from Michigan patients have identified differences in resistance frequencies when compared to regional, state and national estimates reported by NARMS. Indeed, differential selective pressures as well as varying exposures and risk factors in distinct geographical locations can impact circulating strain populations and resistance frequencies.

Multiple genotyping tools have been used to characterize *C. jejuni *. Multilocus sequencing typing (MLST), for instance, classifies strains based on seven conserved housekeeping genes that are useful for epidemiological, evolutionary and ecological studies [9]. Our prior study demonstrated that specific multilocus sequence types (STs) predominated among clinical cases and were correlated with antibiotic resistance phenotypes [6]. More recently, however, the CDC has recommended the use of whole-genome sequencing (WGS) for characterizing *C. jejuni* and other foodborne pathogens [10]. WGS has proven to be useful for public health by aiding in outbreak detection and epidemiological investigations, defining emerging strain types and outbreak sources, and detecting common genes linked to antibiotic resistance and virulence.

The use of WGS can also be helpful for redefining the population structure of *Campylobacter* spp. and identifying new associations with specific hosts or environments. A prior study using MLST, for instance, showed that some *C. jejuni* STs and clonal complexes (CCs) were correlated with certain hosts (e.g. cattle or chicken) more so than the geographical location [11]. These lineages were defined as ecological specialists, which are typically restricted to one host [12] and can thrive in certain agricultural environments due to natural selection [13]. Varying frequencies of specialist lineages have been observed in infected humans across locations [14], highlighting the importance of geography-specific exposures. By contrast, ecological generalists have also been identified [15], which were suggested to have a broader distribution and are capable of surviving in multiple environments and hosts [12]. Although the generalist lineages tend to cause a higher rate of human infections, the disease frequencies for these lineages also vary across geographical locations [9, 16, 17]. Such variation probably reflects the conditions that promote bacterial survival and expansion in the respective niche along with unique risk factors and exposures that influence *Campylobacter* acquisition and infection in humans.

To better understand how genetic variation in *C. jejuni* is linked to human infections, we applied WGS to 214 clinical strains recovered from patients with campylobacteriosis in Michigan. We aimed to comprehensively define the diversity of the strain population using pangenomic approaches and identify relationships between lineages and epidemiological factors. We also sought to define the distribution and diversity of clinically important antibiotic resistance genes (ARGs), which can promote the identification of discriminatory markers within the pangenome that are correlated with resistance phenotypes. Understanding how geographical variation impacts strain traits and distributions is important to direct the implementation of site-specific public health interventions that can decrease the incidence of human infections.

## Methods

### Bacterial strains

An active surveillance system was developed in collaboration with the Michigan Department of Health and Human Services (MDHHS) to recover 217 *C *. *jejuni* isolates from patients with campylobacteriosis between 2011 and 2014 as described [6]. Isolates were grown at 37 °C on tryptone soy agar (TSA) supplemented with 5 % sheep blood in microaerophilic conditions and DNA was isolated using the Qiagen DNeasy Kit (Qiagen) as described [7].

### Whole-genome sequencing and bioinformatic analysis

DNA libraries were constructed with the Nextera XT library prep kit (Illumina) and sequenced on the MiSeq (Illumina) with 2×250 bp reads at the Michigan State University (MSU) Research Technology Support Facility or the Michigan Department of Agriculture and Rural Development.

WGS analyses were adapted from those described for Shiga toxin-producing *E. coli* [18, 19]. Raw reads were trimmed using Trimmomatic v0.36 [20] with a four-base sliding window, minimum PHRED score of 15 and length of 35. FastQC v4.10.1 (https://www.bioinformatics.babraham.ac.uk/projects/fastqc) was used to confirm read quality and *de novo* genome assembly was performed using Spades v3.15.2 (kmers 21, 33, 55, 77, 99 and 127) with error correction to minimize mismatching [21]. To confirm genome quality, QUAST [22] was used with reference genome NC_002163.1 available through the NCBI and MultiQC v1.10.1 [23]. Prokka v1.14.6 allowed for the annotation of each genome using ‘usegenus’ with parameters for *Campylobacter* [24]. Genomes were filtered by the number of contigs, assembly size, GC content and predicted number of coding genes.

Three of the 217 sequenced strains were excluded due to poor quality, leaving an assembled set of 214 contiguous sequences for analysis. These 214 strains were included in most downstream analyses. In addition, a subset of 87 strains were classified as being specific to Michigan since they were from cases reporting no history of travel outside the state in the past month. It was therefore assumed that these patients acquired *C. jejuni* while in Michigan.

### Pangenome and phylogenetic evolutionary reconstructions

The Roary pangenome pipeline [25] was used with -i (blastp of 95 %) and the -e parameter (PRANK [26] aligner 170427) to create a multi-FASTA alignment of core genes. Core gene sequences were concatenated into a single alignment, which was used to generate a maximum-likelihood (ML) phylogeny with RAxML [27]. Four gamma categories for rate heterogeneity were used (parameters -m GTRGAMMA -N and 100 bootstrap replicates), and the phylogeny was visualized via the Interactive tree of life (IToL) (https://itol.embl.de/). Data plots were generated using ‘Roary plots.py’ (https://github.com/sanger-pathogens/Roary/tree/master/contrib/roary_plots) along with the gene presence and absence matrix. A neighbour-net tree based on the 615 core genes was also reconstructed using SplitsTree v4.17.0 [28]; the pairwise homoplasy index (PHI) was calculated to detect recombination between genomes [29]. Finally, the Microbe Genome Atlas pipeline [30] was utilized to determine the average nucleotide identity (ANI). This pipeline utilizes FastANI [31] to estimate the ANI and generate a pairwise assessment of all orthologous genes across the queried strains.

### *In situ* molecular typing

Contigs were queried for ARGs with blast-based programs, ABRicate (https://github.com/tseemann/abricate) and StarAMR [32]. The Resfinder 4.0 [33] and PointFinder [34] databases were also used to identify genes and point mutations previously shown to be important for resistance. Antibiotic resistance phenotypes for tetracycline, ciprofloxacin, phenicol and the macrolide, lincosamide and ketolide (MLK) antibiotics were determined in our prior studies using broth microdilution assays [6, 7]. These resistance phenotypes were compared to the genotypic data to evaluate concordance. Multidrug resistance (MDR) was defined as phenotypic resistance to three or more antibiotic classes, while strains were classified as having genotypic MDR if they harboured ARGs or mutations conferring resistance to three of more antibiotic classes. Contigs were also queried for MLST loci to assign STs and CCs through StarAMR [32] and mlst (https://github.com/tseemann/mlst), which interface with the PubMLST database (https://pubmlst.org/) [35], while PlasmidFinder [36] was used to identify plasmids within the genomes.

### Data analysis

CCs and STs were defined as generalist and cattle- or chicken-specialist lineages based on data generated in prior studies [12, 13, 37], which examined a global collection of *C. jejuni *. Within some CCs, such as the ST-21 CC, Cobo-Díaz *et al*. [37] demonstrated that certain STs were associated with specific hosts; hence, these designations were used for host source assignments in our dataset (Table S1). If specific STs within a given CC were not evaluated or found to be linked to a reservoir host in subsequent studies, then the associations between CCs and host sources identified by Sheppard *et al*. [12] were used.

For the epidemiological analyses, demographic, exposure and clinical data were previously extracted from the Michigan Disease Surveillance System (MDSS) [7] and managed using Microsoft Access and Excel. Univariate analyses were conducted to identify factors associated with specific lineages identified in the pangenome analysis. Odds ratios (ORs) and their 95 % confidence intervals (CIs) were calculated to describe the magnitude of each association. Fisher’s exact test was used for variables with small sample sizes (fewer than five per cell), whereas the Mantel–Haenszel (MH) chi-square test evaluated trends over time and the chi-square test for equal proportions compared frequencies between groups. Variables with a *P*-value of ≤0.20 in the univariate analysis were included in the multivariate analysis. Stepwise logistic regression was performed with forward selection to identify predictors of infections caused by specific pangenomic lineages. The Hosmer–Lemeshow Goodness-of-Fit test (*P*>0.05) provided support for each model. Adjusted ORs and the Wald chi-square test with 95 % Wald confidence limits were calculated to identify significant predictors. All analyses were performed in SAS 9.4 (SAS Institute), while Epi Info 7 (https://www.cdc.gov/epiinfo/index.html) was used to confirm OR estimates and their 95 % CIs.

Lastly, the county of residence was used to determine the longitude and latitude of the public health departments located within each county for mapping; MicroReact [38] was used for the phylogeographical and temporal analyses along with the R packages ape [39] and ggtree [40].

## Results

### Specific *C. jejuni* genotypes predominate in Michigan patients

Among the 214 *C *. *jejuni* strains with adequate sequencing data, the number of contigs ranged from 13 to 332 per genome with a median of 85 (Table S1). The *N*_50_ values ranged between 3000 and 332 500 base-pair sequences (bps), while the assembly size ranged from 1 431 867 to 1 889 890 bps with an average GC content of 30.5 %. Genome completeness, a measure of the fraction of the genome that is assembled as compared to a reference genome [22], ranged from 76.0 to 98.1 % with a median of 93.2 % and average of 92.4 %.

Analysis of the seven MLST loci sequences identified 85 distinct STs that varied in frequency among the 214 strains (Fig. S1A). Two novel STs (12343 and 12344) with unique allele combinations were identified. In all, 54 (63.5 %) of the 85 STs were represented by only one strain, whereas 11 STs were represented by five or more strains each, accounting for almost half (49.1 %; *n*=105) of the 214 strains. STs 353 (*n*=18, 8.4 %), 982 (*n*=16, 7.5 %), 45 (*n*=12; 5.6 %) and 50 (*n*=11, 5.1 %) predominated (Fig. S1B). Only 182 strains, however, could be assigned to one of the 21 predefined CCs. The most common CC identified was the ST-21 CC (*n*=46, 21.5 %) followed by the ST-353 (*n*=36; 16.8 %), ST-45 (*n*=15; 7.0 %), ST-48 (*n*=14; 6.5 %), ST-257 (*n*=12; 5.6 %) and ST-206 (*n*=9; 4.2 %) CCs.

### Classifying strains by host source reveals the importance of specialist lineages

The ST and CC designations were used to assign strains to a predefined host source based on relationships identified in prior studies (Table S1) [12, 13, 37]. As noted, the predefined specialist lineages were linked to either cattle or chicken, whereas the generalist lineages were associated with multiple hosts and environments [12]. Among the 182 strains with CC assignments, 173 (95.1 %) could be assigned to a predefined host source; 41 strains were unassigned (Fig. S1C). Of these 173 strains, most (*n*=93; 53.8 %) were classified as chicken specialist lineages followed by cattle specialists (*n*=34; 19.7 %) and generalists (*n*=46; 26.6 %).

The 93 strains classified as chicken specialists were diverse belonging to eight CCs. The ST-353 CC (*n*=36; 38.7 %) predominated followed by the ST-45 (*n*=14; 15.1 %), ST-257 (*n*=12; 12.9 %), ST-21 (*n*=11; 11.8 %), ST-464 (*n*=8; 8.6 %), ST-607 (*n*=7; 7.5 %), ST-354 (*n*=3; 3.2 %) and ST-443 (*n*=2; 2.2 %) CCs. The cattle specialist lineages were less diverse, representing five CCs, with most strains belonging to the ST-21 (*n*=23; 67.7 %) and ST-61 (*n*=5; 14.7 %) CCs. Two strains were also classified as cattle specialists in each of the following CCs: ST-22 (5.9 %), ST-42 (5.9 %) and ST-403 (5.9 %). Because some STs within CC-21 were previously classified as either generalists or specialists [37], these classifications were used herein. All 11 of the CC-21 chicken specialists belonged solely to ST-50, while the CC-21 cattle specialists represented ST-982 (*n*=16), ST-8 (*n*=4) and ST-806 (*n*=3). Among the 46 strains classified as generalists, most belonged to the ST-48 CC (*n*=14; 30.4 %) or the ST-21 CC (*n*=12; 26.1 %), which were all classified as ST-21. Generalists were also represented by the ST-206 (*n*=9; 19.6 %), ST-52 (*n*=7; 15.2 %), ST-282 (*n*=3; 6.5 %) and ST-283 (*n*=1; 2.2 %) CCs.

### Pangenome analysis uncovers core genes and a diverse strain population

Among the 214 genomes, 8781 unique genes were identified, with 615 being classified as core genes due to their presence in ≥99 % (*n*=211) of the strains (Fig. S2). These core genes represent only 7.0 % of the total pangenome. Another subset of 357 genes was classified as soft-core genes, which are defined as being present in 95–99 % of all genomes or between 203 and 211 of the strains. When combined, the core and soft-core genes comprised 11.1 % of the *C. jejuni* pangenome and were detected in at least 203 strains. Most genes, however, were defined as accessory genes comprising 88.9 % of the pangenome, with 1169 loci representing shell genes and 6640 loci comprising cloud genes. The shell genes were defined based on their presence in 15–95 % of strains, whereas the cloud genes were found in <15 % of the 214 strains.

**Fig. 1.**
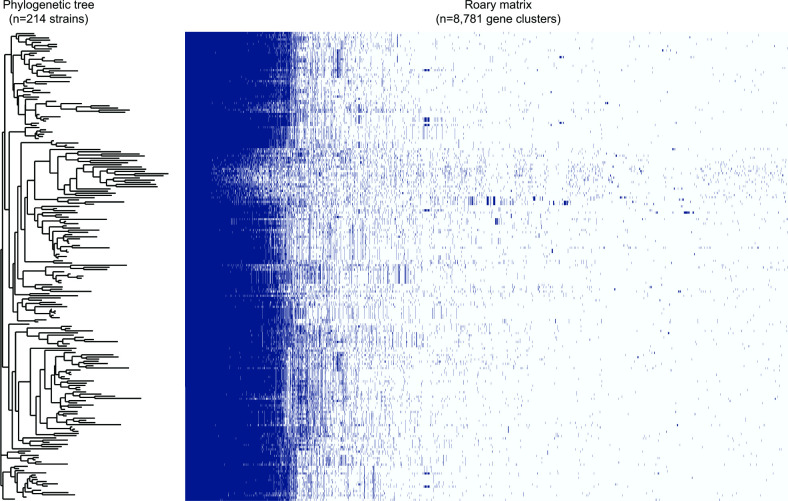
Maximum-likelihood phylogeny of 214 *Campylobacter jejuni* strains reconstructed using the 615 core-gene sequences (550 736 bp) identified in the pangenomic analysis and rooted at the midpoint. The heat map shows the presence (dark blue shading) or absence (white shading) of the 8781 unique genes (arranged in columns) per strain based on placement in the phylogeny.

Alignment of the 615 core genes (550 736 bp) in the 214 assembled and annotated genomes and reconstruction of a ML phylogeny indicated a diverse *C. jejuni* population with most strains occurring in small clusters located on separate branches (Fig. 1). The clusters within the phylogeny are grouped based on the presence or absence of the 615 genes. A diverse array of gene clusters was observed across the phylogeny with descendant and distant lineages showing evidence for gene loss and gain. It is notable that the distinct cluster of about ten strains in the middle of the phylogeny lacked multiple genes.

Substantial diversity was also observed in the number of protein-coding genes per genome with a range between 1387 and 2008. Most of these genes were strain specific, as 2699 singleton genes belonged to only one strain comprising 30.7 % of the pangenome (Fig. S3). Analysis of the ANI values across genomes indicated a range of 96.7–99.9 % with an average of 98.4 % and median of 98.5 %. Interrogation of plasmid sequences associated with each genome detected no plasmids in any of the 214 strains. However, this finding is probably due to the methodology used rather than the true absence of these mobile genetic elements within these genomes.

### Core-gene alignment of clinical *C. jejuni* strains resolves three clusters

**Fig. 2.**
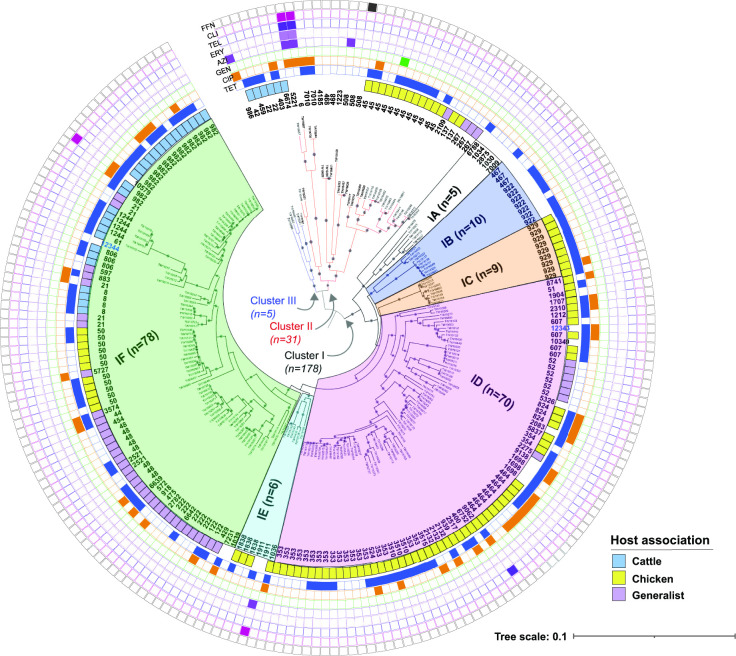
Maximum-likelihood phylogeny based on the 615 core genes and rooted at the midpoint between the two longest branches. Sequence Clusters I–III are labelled, while the Cluster I subclusters (IA–IF), which grouped with >80 % bootstrap support, are shaded with different colours. Multilocus sequence types (STs) are noted at the end of each branch along with the antibiotic resistance phenotypes and host association (if known). New STs are indicated in blue text. The coloured squares represent phenotypic resistance to the following: phenicol, FFN (black); clindamycin, CLI (fuschia); telithromycin, TEL (indigo); erythromycin, ERY (light purple), azithromycin, AZI (dark purple); gentamicin, GEN (green); ciprofloxacin, CIP (orange); or tetracycline, TET (blue).

The ML phylogeny based on the 615 core genes resolved three distinct sequence clusters that each grouped together with >80 % bootstrap support (Fig. 2). Some strains within each cluster were highly similar and grouped together with 100 % bootstrap support, while others were found on distinct branches. Cluster I was the largest and most diverse group containing 178 (83.2 %) strains representing 68 distinct STs belonging to 14 CCs; strains within Cluster I could be further divided into six related subclusters, IA–IF, with high bootstrap support (94–100 %). Cluster II comprised 31 (14.5 %) strains representing 13 STs within five CCs, whereas Cluster III contained five (2.3 %) strains belonging to four STs within two CCs. The proportion of the Cluster I subclusters, IA-IF, also varied, with subclusters IF (*n*=78; 43.8 %) and ID (*n*=70; 39.3 %) predominating among the 178 Cluster I strains. Fewer strains were classified as subclusters IA, IB, IC and IE, ranging from five to ten strains per group.

Within this rooted core-gene phylogeny, all strains with the same STs grouped together within the same cluster. Several STs within subclusters ID or IF, however, were located on distinct branches, highlighting some discordance between the MLST genotypes and the core gene lineages. Two distinct groups of ST-353 and ST-607 strains, for example, were identified within subcluster ID. The same was true for subcluster IF, which contained ST-21 strains on several different branches.

To confirm the population structure displayed in the rooted phylogeny (Fig. 2), an unrooted ML phylogenetic tree was reconstructed based on the 615 core genes. While all three clusters could be differentiated, some additional discrepancies were observed in the clustering of certain STs and CCs within Cluster I (Fig. S4). For instance, the 13 STs belonging to the ST-353 CC mapped to three separate clusters, while strains belonging to the ST-257, ST-52 and ST-48 CCs were each found in two clusters and across subclusters IC–IF. In most cases, however, the strains with the same STs and CCs grouped together within the same cluster and/or subcluster.

### Some resistance phenotypes are concentrated together within the phylogeny

Although the proportion of strains with phenotypic resistance to any of the antibiotics tested did not significantly differ across the clusters (MH chi-square *P*=0.48), the rooted phylogeny shows that some phenotypes are concentrated within some clusters (Fig. 2). Indeed, 81.5 % of the 135 resistant strains grouped within subcluster IF (*n*=48; 35.6 %), subcluster ID (*n*=46; 34.1 %) and Cluster II (*n*=16; 11.9 %). Moreover, two of the four MDR strains with resistance to ciprofloxacin and MLK antibiotics grouped together within Cluster II (100 % bootstrap support) despite representing different STs (6 and 5221). The remaining two MDR strains, however, had slightly different resistance profiles, representing STs 9062 and 10579 belonging to subclusters ID and IF, respectively.

Although strains with ciprofloxacin resistance mapped throughout the phylogeny, any ciprofloxacin resistance was significantly more common within subcluster ID as compared to all other lineages combined (OR: 2.3; 95 % CI: 1.20–4.50). Notably, 23 (32.9 %) of the 70 subcluster ID strains were phenotypically resistant to ciprofloxacin, representing almost half (47.9 %) of the 48 ciprofloxacin-resistant strains (Table S2). Even though 20.5 % of the 78 subcluster IF strains were resistant to ciprofloxacin, this lineage was not significantly more likely to be resistant than all others combined (OR: 0.8; 95 % CI: 0.43–1.65). By comparison, the distribution of tetracycline resistance was more widespread across the phylogeny, even though all nine subcluster IC strains were resistant (Fisher’s exact test; *P*=0.005). Despite a greater proportion of strains with tetracycline resistance in subclusters ID (34.2 %) and IF (36.7 %), neither had significantly more resistance than the other lineages combined. It is also notable that strains within Cluster II were less likely to be resistant to tetracycline compared to the other lineages (OR: 0.4; 0.20–0.96), yet the association was not significant (*P*=0.06).

### Genotypic resistance profiles correlate with resistance phenotypes

**Fig. 3.**
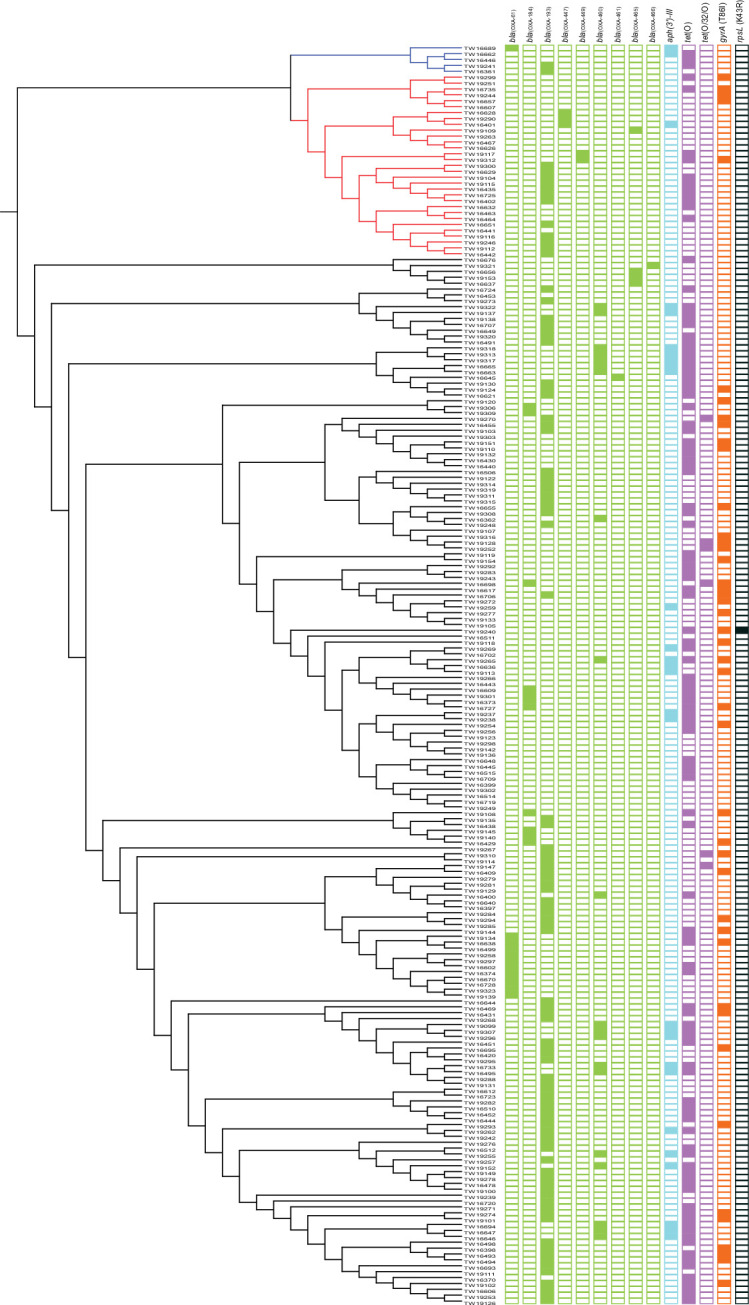
Genotypic resistance profiles in the 214 *Campylobacter jejuni* genomes shown within the maximum-likelihood phylogeny constructed using the 615 core genes. Coloured squares indicate the strains with a gene or point mutation conferring resistance to the following classes: green, β-lactams; blue, aminoglycosides; purple, tetracyclines; orange, quinolones and fluoroquinolones; and black, MLK antibiotics. Gene names and mutations are indicated at the top, and the coloured branches highlight the three sequence clusters.

Fourteen unique ARGs encoding resistance to the β-lactam, tetracycline and aminoglycoside antibiotic classes were detected within the 214 genomes (Fig. 3). At least one ARG was detected in most (*n*=191; 89.3 %) strains, while 108 (50.5 %) strains had more than one resistance gene. ARGs encoding β-lactamases, which confer resistance to the β-lactam antibiotics, were the most common (*n*=148; 77.5 %) and diverse encoding nine different β-lactamase (*bla*) genes. *bla*_(OXA-193)_ predominated in 63.5 % (*n*=94) of the 148 strains followed by *bla*_(OXA-460)_ in 13.5 % (*n*=20) of strains. None of the strains carried more than one *bla* gene. Tetracycline resistance genes encoding ribosomal protection proteins were found in 55.6 % (*n*=119) of strains. Most of these strains harboured *tet*(O) (*n*=112; 94.1 %), though seven (3.3 %) had *tet*(O/32/O), a mosaic chimeric gene that evolved by recombination between *tet*(O) and *tet* [32, 41]. Only 28 (13.1 %) of the 214 strains possessed *aph(3')-III*, which confers resistance to aminoglycosides.

Several point mutations linked to resistance were also detected. A well-defined Thre-86-Ile amino acid substitution in the gyrase A gene (*gyrA*) was found in the quinolone resistance-determining region [42] in 44 (20.6 %) genomes (Fig. 3). A mutation in *rpsL* (K43R), which was previously linked to streptomycin resistance [43], was also found in a ST-9062 strain (TW19240) belonging to the ST-353 CC within subcluster ID (Fig. 2). This strain was genotypically classified as MDR given that it also possessed *tet*(O) and the *gyrA* mutation conferring tetracycline and fluoroquinolone resistance, respectively.

In all, 40 (18.7 %) strains were genotypically classified as MDR because ARGs or SNPs conferring resistance to more than three antibiotic classes were detected. Most (*n*=20; 50.0 %) of these MDR strains had two genes encoding tetracycline and β-lactam resistance plus the *gyrA* mutation for fluoroquinolone resistance, while 18 (45.0 %) strains had three ARGs for the tetracyclines, β-lactams and aminoglycosides. Another ST-2132 strain (TW19265) of the ST-353 CC harboured genes for tetracycline, β-lactam and aminoglycoside resistance and also contained the *gyrA* mutation for resistance to fluoroquinolones. Genotypic MDR was more common in certain lineages relative to what was observed for phenotypic resistance, though susceptibility to the β-lactams was not evaluated previously [6, 7]. Indeed, inclusion of β-lactam resistance genes in the genotypic profiles indicates that most strains with genotypic MDR grouped within subcluster IF (*n*=22; 55.0%). Most (*n*=16; 72.7 %) of these MDR strains belonged to the ST-21 CC, of which seven (43.8 %) belonged to the cattle specialist ST-982 and five to the chicken specialist ST-50 (31.3 %).

Importantly, a strong correlation was observed between ARGs detected in the genomes and the previously determined phenotypic resistance profiles for the MLK antibiotics as well as ciprofloxacin, tetracycline and chloramphenicol [6, 7] (Table 1). The sensitivity and specificity of WGS screening for ciprofloxacin resistance was 0.90 and 0.99, respectively, while the sensitivity and specificity for tetracycline resistance were both 0.98. Most discrepancies involved aminoglycoside resistance, which had a 12.7 % major error. No ARGs were detected for the MLK antibiotics.

**Table 1.** Major and minor error between antibiotic resistance genotyping as determined by whole-genome sequencing (WGS) and antibiotic susceptibility testing (phenotyping); the sensitivity and specificity of WGS for predicting antibiotic resistance phenotypes is also shown

Antibiotic	No. with resistance phenotype	No. with resistance genotype and phenotype	Very major error*	No. of susceptible phenotypes	No. of susceptible genotypes and phenotypes	Major error†	Sensitivity	Specificity
Ciprofloxacin	48	43	5 (2.4 %)	166	165	1 (0.5 %)	0.896	0.994
Tetracycline	120	117	3 (1.4 %)	95	93	2 (0.9 %)	0.975	0.979
Gentamicin	1	0	1 (0.5 %)	213	186	27 (12.7 %)	−	0.873
Chloramphenicol	1	0	1 (0.5 %)	213	213	0	−	1
MLK antibiotics	6	1	5 (2.4 %)	208	208	0	0.167	1

*Very major error occurs if a phenotypically resistant isolate is genotyped as susceptible. In other words, this error is the failure to detect phenotypic resistance using genotypic methods.

†Major error occurs if a phenotypically susceptible isolate is genotyped as resistant. In other words, the genotypic tests predict there is resistance when there is none.

MLK, Macrolide, lincosamide, ketolide antibiotics.

### Identification of temporal changes and epidemiological associations by cluster

The distribution of lineages identified in the pangenomic analysis differed significantly over the 4 year period (MH chi square *P*=0.04). Specifically, the proportion of Cluster I strains increased from 70.6 % in 2011 to 88.5 % in 2014 relative to strains belonging to Clusters II and III (MH chi square *P*=0.03) (Fig. S5). Among the subclusters, a significant change was only observed for subcluster ID, which increased from 23.5 % in 2011 to 41.0 % (*n*=32) in 2014 (MH chi square *P*=0.02). The proportion of subcluster IF strains remained stable across the 4 year period (MH chi square *P*=0.82).

Children under 9 years of age were significantly more likely to be infected with Cluster I strains relative to adults between 19 and 40 years of age; however, no difference was observed when comparing these children to all other age groups combined (Table S3). In the univariate analysis, significant associations were also observed for travel history and rural residence. Among the 61 cases reporting travel in the past month, 55 (90.2 %) had Cluster I infections compared to 68 of the 87 cases (78.2 %) who had not travelled (OR: 2.6; 95 % CI: 0.96–6.83). Thirty-five (23.3 %) of these patients reported travelling in the USA, whereas 26 (17.3 %) travelled internationally; two individuals did not specify. Notably, patients reporting international travel were significantly more likely to have Cluster I infections (*n*=26; 96.2 %) than Clusters II and III infections (*n*=1; 4.0 %) (Fisher’s exact test; *P*=0.04). No association was observed for domestic travel (*P*=0.34). Patients with Cluster I infections were also more likely to reside in rural counties (OR: 2.4; 95 % CI: 1.02–5.62) and counties with high cattle densities (OR: 2.8; 95 % CI: 0.88–9.01), though the latter was not significant. For the clinical variables examined, patients with a Cluster II or Cluster III infection (21.4 %) were twice as likely to be hospitalized than those with Cluster I infections (41.4 %; OR: 2.6; 95 % CI: 1.13–5.95). No associations were observed between the pangenomic cluster and specific clinical symptoms (e.g. abdominal pain, bloody stool or body aches) as well as sex, race, season, well water use and recent poultry consumption.

To identify predictors of Cluster I infections relative to Clusters II and III, multinomial logistic regression was applied to 133 patient records with complete data. Potential confounders such as age, race and sex as well as notable variables identified in the univariate analysis (e.g. residence, recent animal contact and international travel) were included in the model. Controlling for these factors identified rural residence to be the only significant predictor of Cluster I infections (adjusted OR: 4.2; 95 % CI: 1.36–13.22). The Hosmer–Lemeshow Goodness-of-Fit test was not significant (*P*>0.40), providing support for the model. No additional associations were observed for phenotypic resistance, cattle density or hospitalization, which were added individually to the model.

### Travel history impacts the diversity of *C. jejuni*

Because recent travel history was associated with Cluster I infections in the univariate analysis, we compared the diversity and population structure of the strains by travel status. For this analysis, the 87 cases reporting no travel in the past month were assumed to have acquired infections while in Michigan. A pairwise genomic comparison of these 87 Michigan-specific strains defined the ANI between orthologous gene pairs to be between 96.7 and 99.9 % with a mean and median of 98.3 and 98.5 %, respectively. Additionally, these genomes were less variable with 6322 genes comprising the pangenome, which is 1.4 times less than the number detected among all 214 strains combined. Roughly 511 core and 501 soft-core genes were identified, which were 1.2 times greater and 1.4 times fewer than in all strains combined, respectively. No difference was observed in the number of coding sequences (CDS) for the 87 Michigan-specific strains (range: 1387–1948; average: 1732) compared to the 214 strains (range: 1387–2008; average: 1741).

**Fig. 4.**
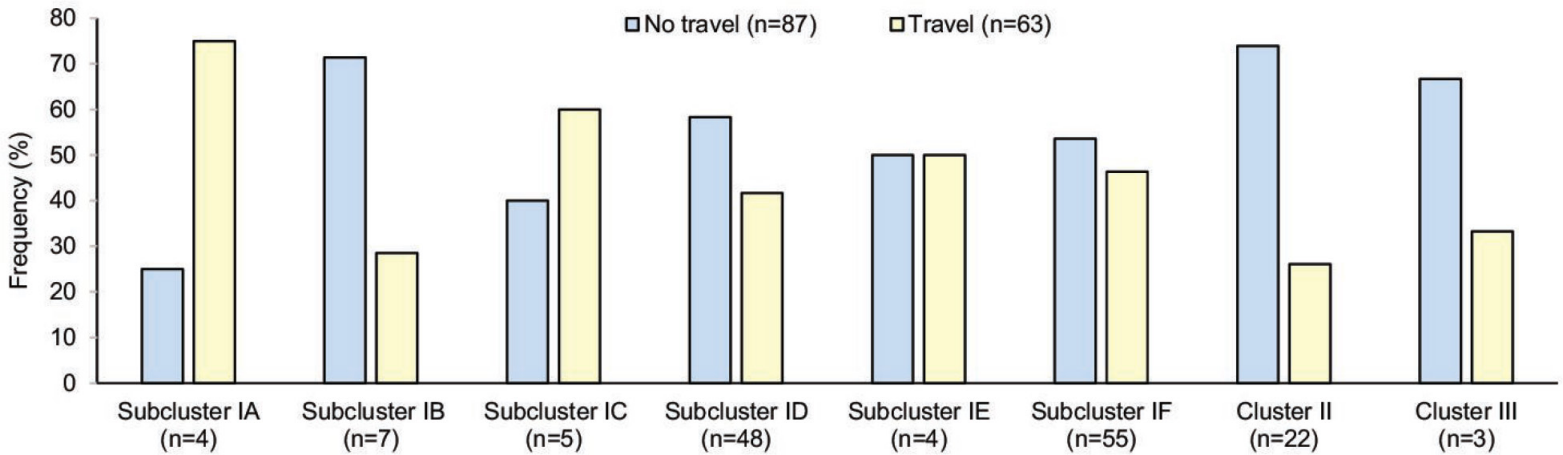
Proportion of pangenomic sequence clusters among the 150 cases reporting travel information. Strains from cases reporting no recent travel (blue) were compared to those reporting domestic or international travel (yellow) in the past month. Those cases who did not travel were assumed to have infections originating from sources or exposures within Michigan. Percentages were calculated using the total number in each lineage as the denominator.

The proportion of pangenomic lineages also differed among those with and without a recent history of travel. Specifically, 53.6 % (*n*=28) of the subcluster ID strains were from cases reporting no travel compared to 41.7 % (*n*=20) among patients with travel (Fig. 4). Similar differences were observed for subclusters IB and IF as well as Clusters II and III, though the sample size is small in some groups. Among the 71 Michigan-specific strains from patients reporting no travel, most (*n*=35; 49.3 %) were classified as chicken specialists. Fewer strains were classified as cattle specialists (*n*=16; 22.5 %) and generalists (*n*=20; 28.2 %), though the distribution was not significantly different by travel status (MH chi square *P*=0.33).

Because 91 cases reported poultry consumption prior to their infection, we also examined the strains from these cases more closely. Regardless of residence type, 42 (46.2 %) of the cases reporting poultry consumption had strains designated as chicken specialists compared to the 24.2 % (*n*=22) and 29.7 % (*n*=27) designated as cattle specialists and generalists, respectively. The difference in these proportions was significant (*P*=0.03). Moreover, 27 of the 42 (64.3 %) chicken specialist strains recovered from cases reporting poultry consumption were specific to Michigan in that the patients lacked a history of recent travel.

### The phylogeography of *C. jejuni* differs across Michigan

Although a significant difference was observed in the frequency of clusters among urban and rural residents (MH chi square *P*=0.05), the MicroReact [38] analysis revealed no clear pattern in the distribution of cases across the 17 counties (https://microreact.org/project/dCGmMiVxreTFjNaqKC68gX-rodrigues-ja-et-al-microbial-genomics-2023). Stratifying by subcluster, however, revealed that the four less prevalent groups (IA, IB, IC and IE) were more common in cases residing in rural areas (*n*=13; 56.5 %) than urban areas (*n*=10; 43.5 %). By contrast, subclusters ID (*n*=38 of 58; 65.5 %) and IF (*n*=42 of 73; 57.5 %) as well as Clusters II/III combined (*n*=27 of 35; 77.1 %) were more common in urban cases. Among these four less common subclusters, seven STs were solely recovered from rural cases and included STs 467 (*n*=1), 922 (*n*=5), 929 (*n*=3), 1030 (*n*=1), 1911 (*n*=2), 2875 (*n*=1) and 7009 (*n*=1). Notably, the three ST-929 strains were predefined as chicken specialist lineages belonging to the ST-257 CC in a prior study [12], while the remaining STs have yet to be linked to a source.

Among the 58 *C. jejuni* strains recovered from rural residents with host source designations, 25 (43.1 %) were chicken specialists, 15 (25.9 %) were cattle specialists and 18 (31.0 %) were generalists. Despite these distributions, no significant difference was observed in the proportion of generalists or chicken- and cattle-associated lineages between urban and rural residents (MH chi square *P*=0.70). Similarly, the proportion of cattle specialists was not significantly different in cases residing in counties with high versus low cattle densities (Fisher’s exact test *P*=0.54). Although 17 of the 20 cattle specialists were from cases in high-density areas, only a subset of counties could be classified by cattle density, which may have reduced our ability to detect a difference.

### Recombination is evident within *C. jejuni* genomes from Michigan

**Fig. 5.**
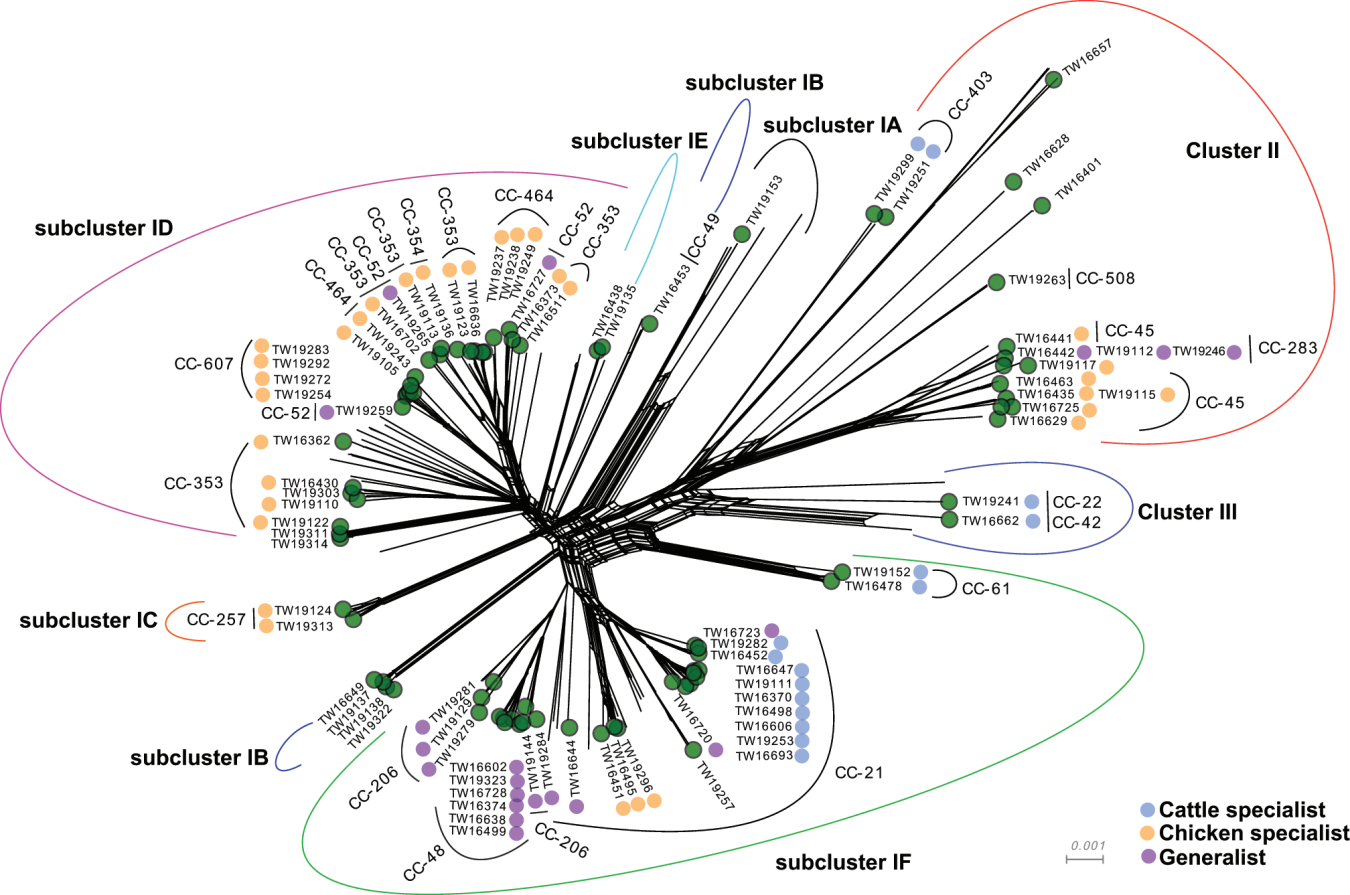
Neighbour-net tree reconstructed using 615 core gene sequences from 214 *C. jejuni* strains overlaid with the pangenomic sequence cluster designations and predefined host associations. The scale is the uncorrected p distance. The clonal complexes (CCs) are indicated within the clusters and subclusters along with the strain accession number (e.g. TW19105). Some lineages are also classified by the predefined host source: cattle (blue), chickens (yellow), and generalists (purple). Only those 87 strains representing the Michigan-specific subset (without a recent history of travel) are labelled.

To determine whether recombination can explain the discrepancies with ST and CC distributions in the rooted versus unrooted phylogenies, a neighbour-net tree was reconstructed using the 615 core gene sequences (Fig. 5). Not surprisingly, significant evidence of recombination was detected across the 214 genomes (PHI; *P*<0.01); the parallelograms between clusters are indicative of reticulate events such as recombination [44]. Although the neighbour-net algorithm differentiated the three sequence clusters, a uniquely bifurcating tree was observed rather than one with central clusters. Unlike the ML phylogenies, subcluster IB was separated into two groups. One group includes strains belonging to ST-467 within the ST-49 CC and the other contains ST-922, which lacked a predefined CC designation.

The tree also demonstrated phylogenetic reticulations and intermingling between the generalist and specialist lineages as well as some clustering by host association (Fig. 4). For example, subcluster ID comprised strains from four chicken associated CCs, 607, 464, 354 and 353, along with one generalist lineage, CC-52, which was represented by three strains located on different branches in the tree. Similar to the ML phylogeny, chicken-associated lineages were also found within Cluster II and subcluster IC. Most (82.4 %, *n*=28) of the 34 cattle-specialist lineages, however, were found in the ST-21 CC within subcluster IF along with most of the generalist lineages. In contrast to the ML phylogeny, the strains representing the ST-61 CC within subcluster IF were separated from the rest of the ST-21 CC by multiple parallel paths that are indicative of recombination.

## Discussion

*C. jejuni* are highly diverse pathogens with the ability to inhabit multiple hosts and environments and cause a range of symptoms in humans. This analysis of 214 *C. jejuni* strains from Michigan patients collected over a 4 year period highlights the extensive genomic diversity within this pathogen population. ANI values, for instance, ranged from 96.7 to 99.9 %, which is consistent with a prior study of 52 *C. jejuni* strains from patients in New Hampshire showing an average ANI value of 98.3 % [45]. The ML phylogeny based on the 615 core genes uncovered three distinct sequence clusters that varied in frequency as well as genotypic and phenotypic traits. The number of core genes was lower than the 1176 observed in the prior study [45], which indicates a more diverse strain population in Michigan. However, only 52 strains were examined in the prior study and all were collected in one year (2017). Larger studies are therefore needed to better estimate the diversity of *C. jejuni *, particularly across and within geographical locations, to define the most common lineages in circulation.

For the most part, strains of identical STs clustered together in the ML phylogeny, though some discrepancies were identified that are probably due to recombination. Consistent with prior studies [12, 45, 46], the population structure of *C. jejuni* was significantly influenced by recombination, which can result in the diversification of specific lineages and the emergence of novel lineages. Two new STs with new allele combinations were identified in Michigan that mapped to different parts of the phylogeny, while two generalist lineages belonging to ST-572 and ST-2782 grouped together within subcluster IF despite having distinct predefined CCs (ST-206 CC and ST-21 CC, respectively). A high degree of relatedness and intermixing of strains from CCs 21, 48 and 206 was also detected, as was observed previously [12, 45, 47].

By contrast, some lineages were divergent, providing support for the previously described recombination barriers between larger generalist populations such as CC-21 [12]. Ecological, mechanistic and adaptive recombination barriers were previously suggested to shape the structure of the *Campylobacter* pangenome [12, 46]. Indeed, some lineages are more widespread in that they have been detected in multiple geographical locations and time periods [12, 48]. In Michigan patients, the common ST-21, ST-353 and ST-45 CCs predominated with 43.9 % of the strains classifying as chicken specialists. This finding illustrates the importance of poultry in this area, though it is probably an underestimate in that 41 of the 214 strains could not be assigned to a predefined CC or host source. This widespread global distribution of some lineages is most likely related to the movement of animals and food products as well as alterations in agriculture practices, as was suggested previously [13]. For example, the CC-353 chicken-specialist lineage and the ST-61 cattle-specialist lineage were also identified in other studies [12, 37, 45]. Since both lineages were part of distinct subclusters in our core gene phylogeny (Fig. 2), however, source attribution studies are needed to determine if these strains are linked to the same hosts in Michigan. The same is true for other lineages such as ST-982, which predominated in this population and grouped together with other cattle specialists (e.g. ST-61) and the ST-21 generalist lineage. Although we previously observed high frequencies of ST-982 strains in cattle recovered during the same time frame [49], we also identified an epidemiological association with chicken contact and well water consumption [7]. Together, these findings suggest that ST-982 may be more of a generalist in Michigan, which is not consistent with the cattle-specialist classification in a global WGS study [37]. Nonetheless, the high degree of genomic relatedness and increased frequency of these ST-982 strains in Michigan also indicates adaptation and proliferation in this environment, which probably has unique agroecological factors relative to other geographical locations. Such differences could contribute to differential exposures or differentially distributed populations of *C. jejuni *; hence, future comparative genomics studies of strains recovered from livestock and patients in different regions are warranted.

The predominance (88.9 %) of genes belonging to the accessory genome is consistent with findings from other studies and further suggests an open pangenome [45, 50]. The observation that 30.7 % of genes were strain specific also highlights the importance of evolutionary processes such as horizontal gene transfer (HGT), which facilitates DNA uptake from other sources. Because *Campylobacter* are naturally competent, they can acquire genetic elements that promote adaptation to a diverse range of niches [51]. Both HGT and clonal expansion have previously been linked to the distribution of resistance determinants within *C. jejuni* [45]. In this study, however, there is little evidence for the clonal expansion of strains carrying specific ARGs. Since genotypic MDR was more common in some related lineages, it is possible that some strain types are more susceptible to HGT and the acquisition of mobile elements containing ARGs. These MDR lineages were also more likely to be cattle or chicken specialists, suggesting that agriculture-associated antibiotic use may select for a resistant strain population that can persist despite subsequent antibiotic exposures. These findings highlight the importance of co-evolutionary processes and demonstrate a widespread distribution of divergent lineages carrying resistance determinants for clinically important antibiotics. Importantly, a high degree of concordance between the presence of ARGs and resistance phenotypes was also observed. Because some discrepancies were identified and no plasmids were detected, however, this discordance illustrates the need for updated databases and plasmid identification tools to enhance genomic classification schemes.

Selective pressures within different environments and hosts are probably contributing to the selection of specific *C. jejuni* strains with certain traits or phenotypes. The distribution of host reservoirs, biogeography and agricultural practices as well as unique risk factors and behaviours in humans are undoubtedly impacting the distribution of these lineages across locations. Indeed, we previously documented differences in resistance frequencies among *C. jejuni* strains recovered in Michigan compared to other states in the USA [6]. Similar differences were also observed in our prior analyses of Shiga toxin-producing *E. coli* [8] and *Salmonella enterica* [52]. Moreover, differences in the distribution of specific *C. jejuni* lineages were identified over time. The significant increase in subcluster ID that primarily contains CC-353, for instance, indicates that a chicken-specialist lineage contributed to an increasing number of human infections over the 4 year period. The finding that chicken-specialist lineages were more common among all Michigan cases and in those reporting poultry consumption further highlights the importance of the chicken reservoir. Additionally, differences in lineage distributions were observed when stratified by urban versus rural residence and travel history, which could be related to the biogeography of the specific sub-lineage niche or host. Such differences could be due to agroecological variation across the state as well as variable risk factors for campylobacteriosis. For instance, age, ethnicity, recent history of travel, livestock exposure and consumption of well water were previously identified as important risk factors [6, 7, 53]. Such findings highlight the importance of continuous monitoring and surveillance efforts in both patients and animal reservoirs.

Collectively, these data highlight how pangenomic approaches can be used to differentiate *C. jejuni *, which is helpful for surveillance efforts and epidemiological investigations to identify potential reservoirs in the absence of source data. These approaches can also facilitate the identification of emerging strain types, new host associations and clinically important ARGs. Surveillance of ARGs in *C. jejuni* strains recovered from different sources is critical to detect changes in resistance frequencies and identify the emergence of new resistant strain types or mutations conferring resistance. Enhancing our understanding of the genomic epidemiology of *C. jejuni* strain populations across locations can also better define risk factors that could guide the development of targeted region-specific prevention strategies.

## Supplementary Data

Supplementary material 1
