# Supplementary material for: Pangenomic analyses of antibiotic-resistant Campylobacter jejuni reveal unique lineage distributions and epidemiological associations
Source: Microb Genom. 2023 Aug 1;9(8):mgen001073. doi: 10.1099/mgen.0.001073 (PMC10483415; doi:10.1099/mgen.0.001073)
Supplement: Supplementary material 1 [file mgen-9-1073-s001.pdf]

Figure S1. The distribution of **A**) multilocus sequence types (STs) and **B**) clonal complexes (CCs) among the 214 *Campylobacter jejuni* isolates as determined by extracting sequences for seven loci used for multilocus sequence typing. **C)** The frequency of strains representing STs and CCs previously linked to a specific host reservoir (e.g., cattle or chicken) or multiple host reservoirs and environments (generalist). Those STs and CCs that have not been previously linked to a host source were classified as “unassigned”.

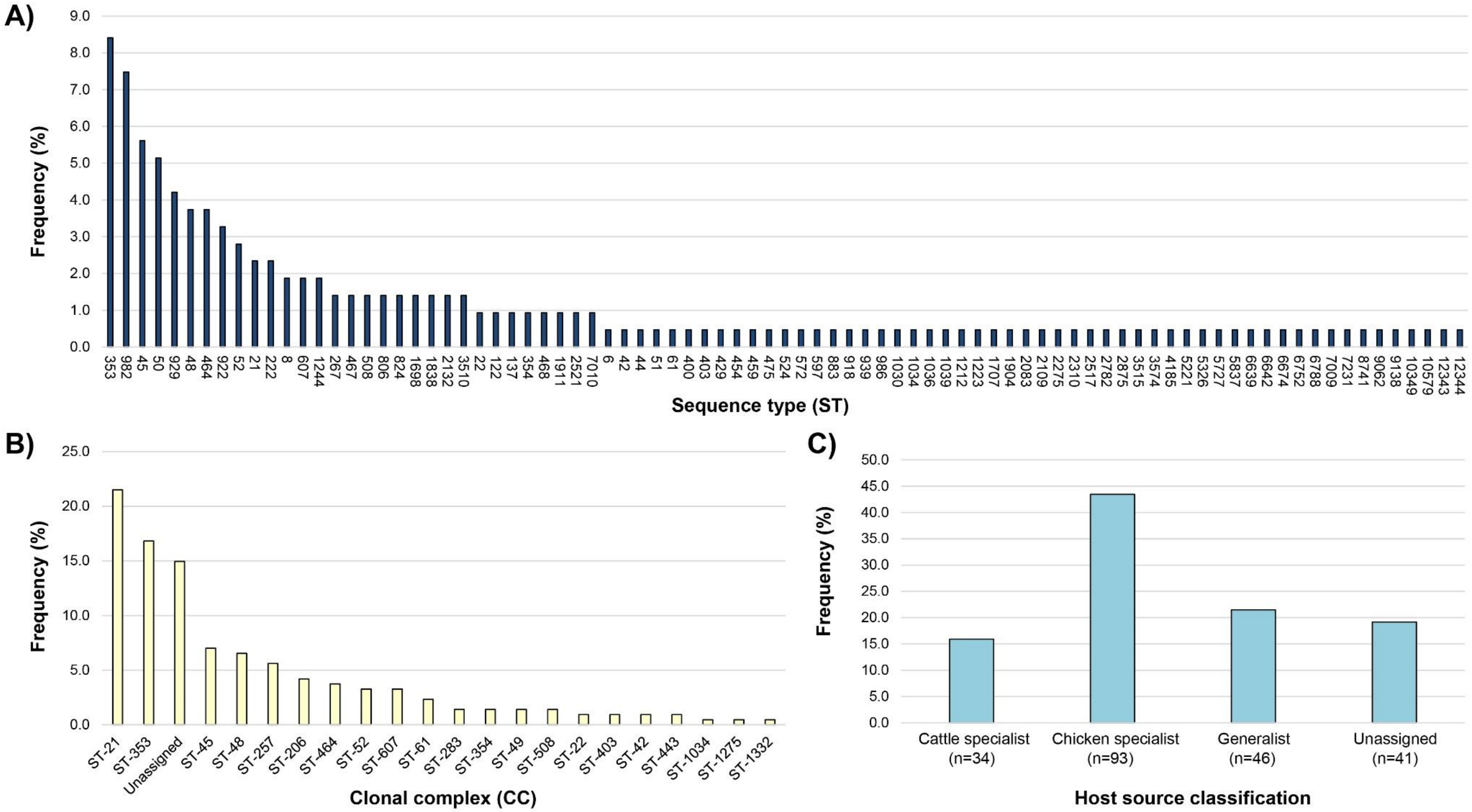
A)
Frequency (%)
Sequence type (ST)
353 982 45 50 929 48 464 922 52 21 222 8 607 1244 267 467 508 806 824 1698 1838 2132 3510 22 122 137 354 468 1911 2521 7010 6 42 44 51 61 400 403 429 454 459 475 524 572 597 883 918 939 986 1030 1034 1036 1039 1212 1223 1707 1904 2083 2109 2275 2310 2517 2782 2875 3515 3574 4185 5221 5326 5727 5837 6639 6642 6674 6752 6788 7009 7231 8741 9062 9138 10349 10579 12343 12344
B)
Frequency (%)
Clonal complex (CC)
ST-21 ST-353 Unassigned ST-45 ST-48 ST-257 ST-206 ST-464 ST-52 ST-607 ST-61 ST-283 ST-354 ST-49 ST-508 ST-22 ST-403 ST-42 ST-443 ST-1034 ST-1275 ST-1332
C)
Frequency (%)
Host source classification
Cattle specialist (n=34)
Chicken specialist (n=93)
Generalist (n=46)
Unassigned (n=41)

Figure S2. Class and number of genes identified among the 8,871 unique genes detected in the pangenomic analysis of 214 *Campylobacter jejuni* strains. Each gene class is represented along with the total number of genes per class and range in the number of strains with a given set of genes. Core genes are defined based on their presence in ≥99% of strains, while the soft-core genes are present in 95%-99% of all genomes. Moreover, the shell and cloud genes were defined based on their presence in 15%-95% of strains and in <15% of the strains, respectively.

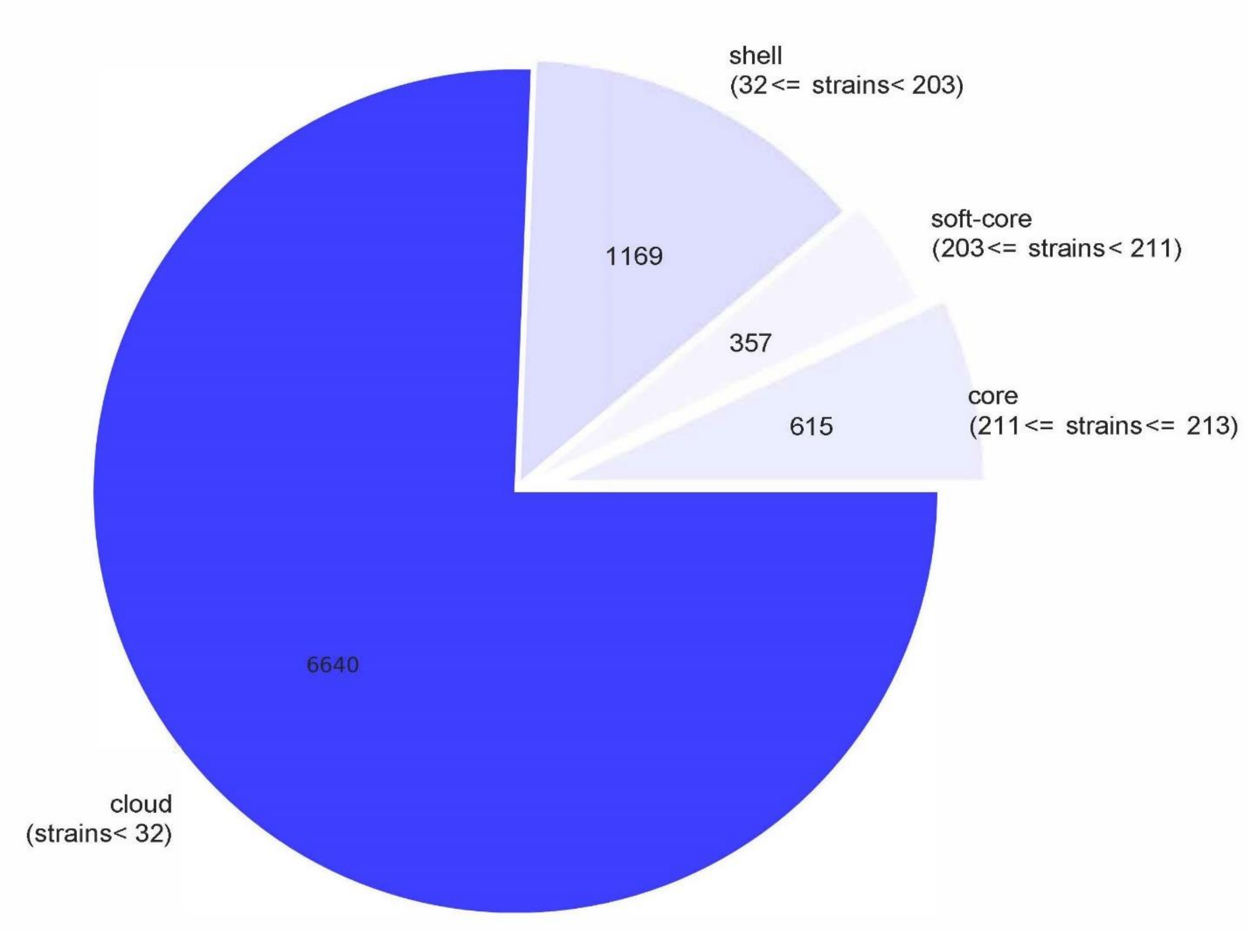

Figure S3. Classification and distribution of the number of genomes for each of the 8,781 unique genes identified among the 214 *Campylobacter jejuni* strains. The 615 core genes are indicated on the right side of the histogram, while the strain-specific genes are shown on the left. A varying number of accessory genes was found across the genomes.

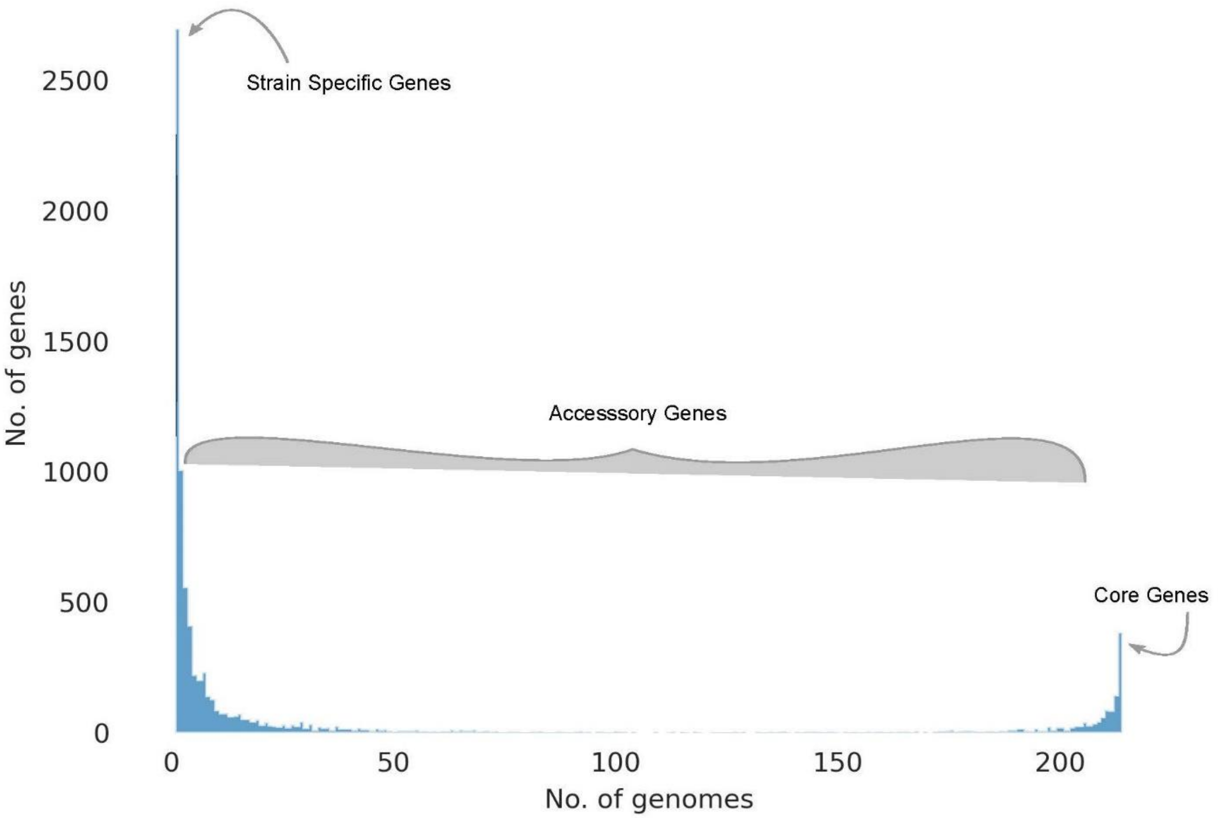

Figure S4. Unrooted maximum likelihood phylogeny based on the 615 core-gene alignment with 100 bootstraps. Strains belonging to the same multilocus sequence type (ST) are collapsed and the nodes are proportional to the number of strains within each ST. Sequence clusters and subclusters are colored, while the clonal complexes (CCs) determined from the ST assignments are annotated at the outer edge. Strains with the same CC designations that span multiple clusters are colored. STs that are differentially placed in the phylogeny relative to the preassigned MLST-based CC designation have black, dotted arrows that point from STs to the pre-assigned CCs.

Note: In this tree, “new2” refers to ST-10579, which was new to this strain population.

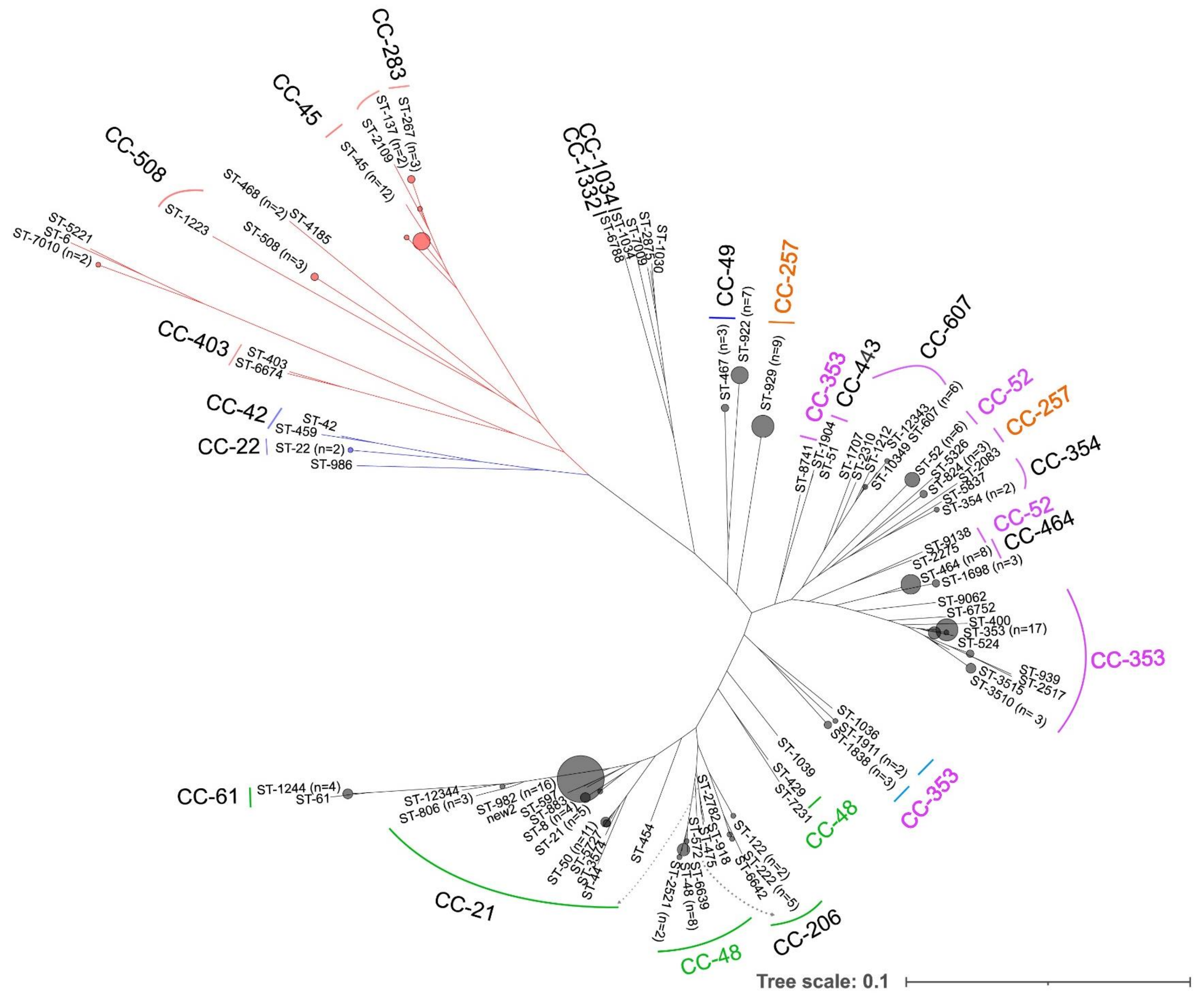
CC-283
ST-267 (n=3)
ST-137 (n=2)
ST-2109
CC-45
ST-45 (n=12)
CC-508
ST-468 (n=2)
ST-4185
ST-1223
ST-508 (n=3)
ST-5221
ST-6
ST-7010 (n=2)
CC-403
ST-403
ST-6674
CC-42
ST-42
ST-459
CC-22
ST-22 (n=2)
ST-986
CC-1034
CC-1332
ST-1030
ST-2875
ST-709
ST-1034
ST-6788
CC-49
ST-467 (n=3)
ST-922 (n=7)
CC-257
ST-929 (n=9)
CC-353
CC-443
CC-607
ST-8741
ST-1904
ST-51
ST-1707
ST-2310
ST-1212
ST-12343
ST-10349
ST-607 (n=6)
CC-52
ST-52 (n=6)
ST-5326
CC-257
ST-824 (n=3)
ST-2083
CC-354
ST-5837
ST-354 (n=2)
CC-52
ST-9138
CC-464
ST-2275
ST-464 (n=8)
ST-1698 (n=3)
ST-9062
ST-6752
ST-400
ST-353 (n=17)
ST-524
CC-353
ST-939
ST-3515
ST-2517
ST-3510 (n= 3)
ST-1036
ST-1911 (n=2)
ST-1838 (n=3)
CC-353
ST-1039
ST-429
ST-7231
CC-48
CC-61
ST-1244 (n=4)
ST-61
ST-12344
ST-806 (n=3)
ST-982 (n=16)
new2
ST-597
ST-883
ST-8 (n=4)
ST-21 (n=5)
ST-50 (n=11)
ST-5727
ST-3574
ST-44
ST-454
CC-21
ST-2782
ST-918
ST-572
ST-475
ST-6639
ST-48 (n=8)
ST-2521 (n=2)
ST-122 (n=2)
ST-222 (n=5)
ST-6642
CC-48
CC-206
Tree scale: 0.1

Figure S5: Number of *Campylobacter jejuni* strains representing the different sequence clusters identified in Michigan patients with infections identified between 2011 and 2014.

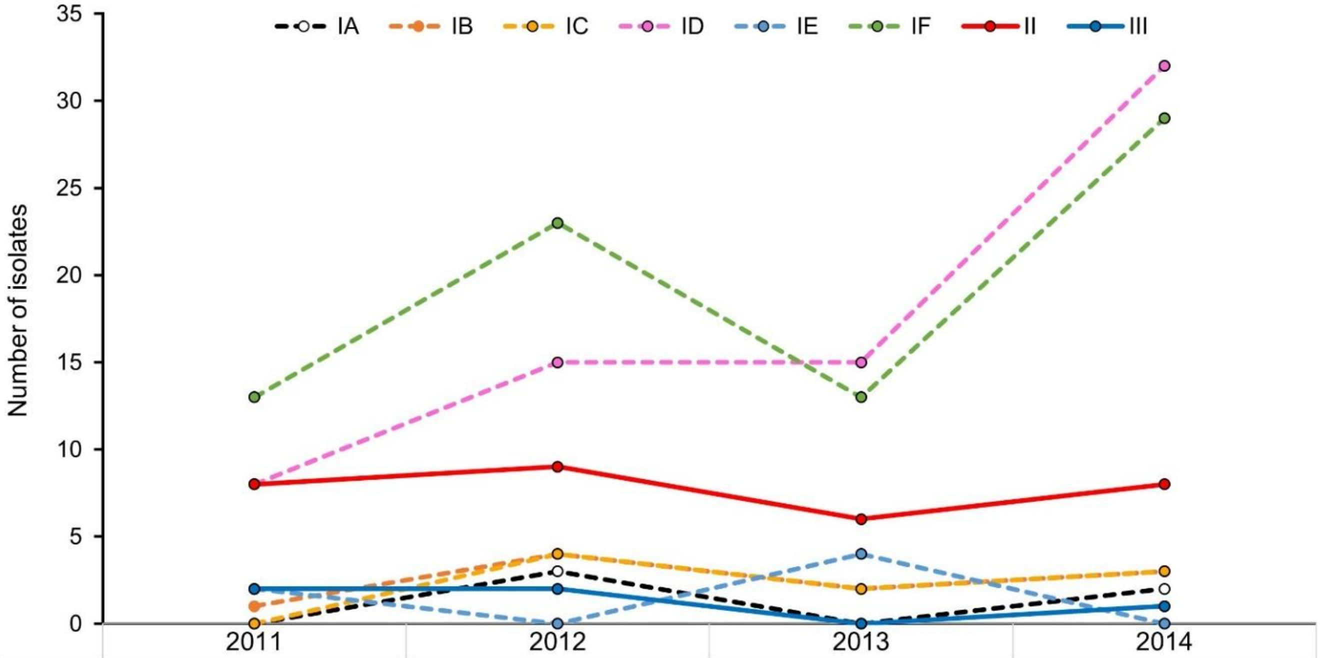

	2011	2012	2013	2014
IA	0	3	0	2
IB	1	4	2	3
IC	0	4	2	3
ID	8	15	15	32
IE	2	0	4	0
IF	13	23	13	29
II	8	9	6	8
III	2	2	0	1

Table S1: Epidemiological data and genomic traits of *Campylobacter jejuni* recovered from humans in Michigan

Strain	BioProject	NCBI ID	Year of isolation	Michigan specific†	Residence Type	bps	bps (Mbp)	GC content	Number of contigs	N50 (Kbp)	L75 (Kbp)	Genome Fraction	CDS	Roary RaXML Cluster**	MLST loci							Sequence type (ST)	Clonal complex (CC)	Host classification	Reference(s) for host classification	Antibiotic resistance phenotype‡
															aspA	glnA	gltA	glyA	pgm	tkt	uncA					
TW16361	PRJNA305291	SAMN04323532	2011		Rural	1608096	1.6	30.5%	36	145.7	60.7	89.9%	1619	III	1	3	6	4	3	3	3	22	ST-22	Cattle	[1]	Susceptible
TW16362	PRJNA305291	SAMN04323533	2011	Yes	Urban	1431867	1.4	31.9%	668	3.0	1.70	77.2%	1533	ID	8	10	2	2	11	12	6	354	ST-354	Chicken	[1,2]	Susceptible
TW16370	PRJNA305291	SAMN04323534	2011	Yes	Rural	1561683	1.6	31.3%	483	5.1	2.8	90.1%	1560	IF	2	1	2	3	2	1	5	982	ST-21	Cattle	[1]	TET
TW16373	PRJNA305291	SAMN04323535	2011	Yes	Rural	1801820	1.8	30.3%	102	58.0	25.4	92.2%	1879	ID	7	17	5	2	13	3	6	3510	ST-353	Chicken	[2]	TET
TW16374	PRJNA305291	SAMN04323536	2011	Yes	Urban	1692248	1.7	30.4%	50	80.7	34.9	94.7%	1724	IF	2	4	1	2	7	1	5	48	ST-48	Generalist	[2]	TET
TW16397	PRJNA305291	SAMN04323537	2011		Urban	1673398	1.7	30.7%	184	16	8.7	93.0%	1755	IF	2	21	5	2	59	1	5	222	ST-206	Generalist	[2]	Susceptible
TW16398	PRJNA305291	SAMN04323538	2011			1652930	1.7	30.5%	42	110.2	51.9	98.0%	1694	IF	2	1	2	3	2	1	5	982	ST-21	Cattle	[1]	CIP
TW16399*	PRJNA305291	SAMN04323539	2011	No		1774103	1.8	30.3%	86	77.1	40	93.6%	1850	ID	7	17	5	2	10	3	6	353	ST-353	Chicken	[1]	Susceptible
TW16400	PRJNA305291	SAMN04323540	2011	No	Urban	1738744	1.7	30.7%	263	13.4	7.4	92.7%	1784	IF	2	469	5	2	59	1	5	6642	ST-206	Generalist	[2]	TET
TW16401	PRJNA305291	SAMN04323541	2011	Yes	Urban	1573680	1.6	31.0%	282	11.9	5.3	83.7%	1646	II	10	2	50	62	91	73	45	468				Susceptible
TW16402	PRJNA305291	SAMN04323542	2011	No	Rural	1643088	1.6	30.4%	38	147.8	54.4	90.2%	1667	II	4	7	10	4	1	7	1	45	ST-45	Chicken	[1]	TET
TW16409	PRJNA305291	SAMN04323543	2011	No	Urban	1709781	1.7	30.4%	51	74.9	40.1	94.6%	1768	IF	6	4	5	2	2	1	5	122	ST-206	Generalist	[2]	CIPTET
TW16420	PRJNA305291	SAMN04323544	2011	No	Urban	1709614	1.7	30.4%	57	77.6	38.1	95.9%	1753	IF	2	1	12	3	2	1	5	50	ST-21	Chicken	[1]	Susceptible
TW16429*	PRJNA305291	SAMN04323545	2011	No		1531629	1.5	31.8%	697	3.3	1.8	82.9%	1650	IE	7	4	5	2	11	3	165	1838	ST-353	Chicken	[2]	TET
TW16430	PRJNA305291	SAMN04323546	2011	Yes	Urban	1637577	1.6	31.4%	684	3.6	1.90	85.0%	1653	ID	8	2	5	53	11	3	1	607	ST-607	Chicken	[1]	TET
TW16431	PRJNA305291	SAMN04323547	2011	No	Urban	1683432	1.7	31.0%	51	124.8	51.9	95.7%	1741	IF	2	1	12	3	2	1	23	3574	ST-21	Generalist	[2]	CIPTET
TW16435	PRJNA305291	SAMN04323548	2011	Yes	Rural	1562684	1.6	30.9%	228	12.7	6.7	87.8%	1563	II	4	7	10	4	1	7	1	45	ST-45	Chicken	[1]	TET
TW16438	PRJNA305291	SAMN04323549	2011	Yes	Urban	1761871	1.8	30.4%	72	57.8	27.8	93.5%	1839	IE	7	84	5	10	119	178	26	1911				TET
TW16440	PRJNA305291	SAMN04323550	2011	Yes	Rural	1598049	1.6	31.5%	692	3.4	1.90	81.9%	1638	ID	8	2	5	53	11	3	1	607	ST-607	Chicken	[1]	TET
TW16441	PRJNA305291	SAMN04323551	2011	Yes	Urban	1425197	1.4	31.7%	584	3.7	2.1	76.0%	1387	II	4	7	10	4	42	7	1	137	ST-45	Chicken	[1]	Susceptible
TW16442	PRJNA305291	SAMN04323552	2011	Yes	Urban	1594062	1.6	30.5%	68	49.7	27.4	87.2%	1595	II	4	7	40	4	42	51	1	267	ST-283	Generalist	[1]	Susceptible
TW16443	PRJNA305291	SAMN04323553	2011	No	Urban	1804604	1.8	30.2%	61	151.6	60.5	92.4%	1892	ID	7	17	5	2	10	3	6	353	ST-353	Chicken	[1]	TET
TW16444	PRJNA305291	SAMN04323554	2011		Rural	1616575	1.6	30.5%	37	140.2	62.3	95.4%	1649	IF	2	1	1	3	2	1	6	8	ST-21	Cattle	[1]	TET
TW16445	PRJNA305291	SAMN04323555	2011		Urban	1725734	1.7	30.3%	92	35.5	23	93.6%	1761	ID	7	17	5	2	10	3	6	353	ST-353	Chicken	[1]	TET
TW16446	PRJNA305291	SAMN04323556	2011		Urban	1574913	1.6	31.3%	367	6.7	3.9	85.2%	1611	III	1	2	3	3	5	9	3	459	ST-42	Cattle	[2,3]	TET
TW16451	PRJNA305291	SAMN04323557	2011	Yes	Rural	1571574	1.6	31.4%	522	4.8	2.7	86.6%	1651	IF	2	1	12	3	2	1	5	50	ST-21	Chicken	[1]	TET
TW16452	PRJNA305291	SAMN04323558	2011	Yes	Rural	1592255	1.6	30.8%	160	18.9	9.4	93.9%	1618	IF	2	1	1	3	2	1	6	8	ST-21	Cattle	[1]	TET
TW16453	PRJNA305291	SAMN04323559	2011	Yes	Rural	1453458	1.5	31.7%	650	3.2	1.9	80.1%	1433	IB	3	1	5	84	11	11	6	467	ST-49			Susceptible
TW16455	PRJNA305291	SAMN04323560	2011	No	Urban	1784198	1.8	30.2%	54	83.1	52	93.9%	1854	ID	166	2	5	72	151	3	1	2310	ST-607	Chicken	[1]	CIPTET
TW16463	PRJNA305291	SAMN04323561	2011	Yes	Urban	1457441	1.5	31.2%	614	3.6	2.0	80.2%	1387	II	4	7	10	4	1	7	1	45	ST-45	Chicken	[1]	Susceptible
TW16464	PRJNA305291	SAMN04323562	2011		Urban	1798755	1.8	30.8%	345	20.9	10.6	91.4%	1848	II	4	7	10	4	1	7	1	45	ST-45	Chicken	[1]	TET
TW16467	PRJNA305291	SAMN04323563	2011		Urban	1570295	1.6	31.1%	328	7.2	4.4	85.7%	1540	II	1	6	60	24	12	28	1	508	ST-508			Susceptible
TW16469	PRJNA305291	SAMN04323564	2011	No	Rural	1658390	1.7	31.1%	328	8.1	5	90.4%	1746	IF	8	1	6	3	2	1	1	44	ST-21	Generalist	[1,2]	CIPTET
TW16478	PRJNA305291	SAMN04323565	2011	Yes	Urban	1536755	1.5	31.2%	357	6.8	3.9	87.3%	1543	IF	1	1	2	2	225	3	17	1244	ST-61	Cattle	[2,3]	TET
TW16491	PRJNA305291	SAMN04323566	2012	No	Rural	1587072	1.6	31.2%	390	6.5	3.8	87.8%	1598	IB	1	1	2	83	2	3	6	922				TET
TW16493	PRJNA305291	SAMN04323567	2012			1726202	1.7	30.9%	311	8.3	5.4	93.7%	1813	IF	2	1	2	3	2	1	5	982	ST-21	Cattle	[1]	CIPTET
TW16494	PRJNA305291	SAMN04323568	2012		Urban	1731020	1.7	30.8%	278	11.4	6	94.1%	1806	IF	2	1	2	3	2	1	5	982	ST-21	Cattle	[1]	CIPTET
TW16495	PRJNA305291	SAMN04323569	2012	Yes	Urban	1654784	1.7	31.0%	231	12.7	7.1	92.3%	1716	IF	2	1	12	3	2	1	5	50	ST-21	Chicken	[1]	TET
TW16498	PRJNA305291	SAMN04323570	2012	Yes	Urban	1669248	1.7	31.0%	161	20.4	13.3	96.1%	1714	IF	2	1	2	3	2	1	5	982	ST-21	Cattle	[1]	TET
TW16499	PRJNA305291	SAMN04323571	2012	Yes	Urban	1622213	1.6	30.8%	112	27	18	93.6%	1652	IF	2	4	1	2	7	1	5	48	ST-48	Generalist	[2]	TET
TW16506	PRJNA305291	SAMN04323572	2012		Rural	1619650	1.6	30.7%	132	21.9	11	91.2%	1665	ID	9	25	2	10	22	3	6	52	ST-52	Generalist	[1]	Susceptible
TW16510	PRJNA305291	SAMN04323573	2012	No	Urban	1605648	1.6	30.6%	80	37.5	23.3	94.7%	1637	IF	2	1	1	3	2	1	6	8	ST-21	Cattle	[1]	TET
TW16511	PRJNA305291	SAMN04323574	2012	Yes	Urban	1704349	1.7	30.7%	233	16.5	7.0	90.8%	1770	ID	7	17	2	2	22	3	6	6752	ST-353	Chicken	[2]	Susceptible
TW16512	PRJNA305291	SAMN04323575	2012	No	Rural	1786822	1.8	30.4%	106	26.8	17.3	94.6%	1868	IF	2	1	1	3	140	3	5	806	ST-21	Cattle	[1]	TET
TW16514	PRJNA305291	SAMN04323576	2012		Urban	1652472	1.7	30.5%	123	30.9	15.1	92.1%	1720	ID	7	17	5	2	10	3	6	353	ST-353	Chicken	[1]	Susceptible
TW16515	PRJNA305291	SAMN04323577	2012		Urban	1732812	1.7	30.5%	169	21	14	92.0%	1810	ID	7	17	5	2	10	3	6	353	ST-353	Chicken	[1]	TET
TW16602	PRJNA305291	SAMN04323578	2012	Yes	Urban	1648108	1.6	30.7%	193	16.7	9.4	92.6%	1671	IF	150	4	1	2	7	1	5	2521	ST-48	Generalist	[2]	TET
TW16606	PRJNA305291	SAMN04323579	2012	Yes	Rural	1781109	1.8	30.3%	43	104.7	62.9	97.4%	1871	IF	2	1	2	3	2	1	5	982	ST-21	Cattle	[1]	TET
TW16607	PRJNA305291	SAMN04323580	2012		Urban	1737897	1.7	31.0%	317	11.3	5.1	83.8%	1823	II	63	34	27	33	45	5	7	6				CIPAZIERYCLITEL
TW16609*	PRJNA305291	SAMN04323581	2012	No		1820022	1.8	30.2%	69	92.4	37	92.2%	1907	ID	7	17	5	2	10	3	6	353	ST-353	Chicken	[1]	TET
TW16612	PRJNA305291	SAMN04323582	2012	No	Rural	1620402	1.6	30.5%	44	98.4	62.3	97.7%	1658	IF	2	1	1	3	2	1	5	21	ST-21	Generalist	[1,2]	Susceptible
TW16617	PRJNA305291	SAMN04323583	2012		Urban	1672136	1.7	30.6%	251	13.5	6.9	91.2%	1679	ID	24	2	2	2	10	3	1	464	ST-464	Chicken	[1]	CIPTET
TW16621	PRJNA305291	SAMN04323584	2012	No	Rural	1649632	1.6	30.4%	71	51	26.4	90.7%	1720	IC	9	2	4	62	4	5	17	929	ST-257	Chicken	[2]	TET
TW16626	PRJNA305291	SAMN04323585	2012		Urban	1649200	1.6	30.5%	54	69.7	30.4	90.4%	1665	II	1	6	60	24	12	28	1	508	ST-508			FFN
TW16628	PRJNA305291	SAMN04323586	2012	Yes	Urban	1588919	1.6	30.4%	36	85.5	56	86.8%	1648	II	10	2	50	62	91	367	45	4185				Susceptible
TW16629	PRJNA305291	SAMN04323587	2012	Yes	Urban	1642157	1.6	30.4%	29	95.7	77.8	90.5%	1672	II	4	7	10	4	1	7	1	45	ST-45	Chicken	[1]	Susceptible
TW16632	PRJNA305291	SAMN04323588	2012		Rural	1613084	1.6	30.5%	45	81.2	43.9	89.3%	1622	II	4	7	10	4	1	7	1	45	ST-45	Chicken	[1]	TET
TW16636	PRJNA305291	SAMN04323589	2012	Yes	Urban	1771863	1.7	30.8%	365	8.1	4.4	88.2%	1739	ID	7	2	5	2	2	3	6	2132	ST-353	Chicken	[2]	Susceptible
TW16637	PRJNA305291	SAMN04323590	2012	No	Rural	1633773	1.6	30.4%	65	53	32.4	89.8%	1688	IA	2	59	4	554	131	24	57	7009				Susceptible
TW16638	PRJNA305291	SAMN04323591	2012	Yes	Rural	1721656	1.7	30.4%	32	113.3	91.4	95.4%	1768	IF	2	4	1	2	7	1	5	48	ST-48	Generalist	[2]	CIPTET
TW16640	PRJNA305291	SAMN04323592	2012		Urban	1701332	1.7	30.4%	58	73.5	39.5	95.0%	1760	IF	2	21	5	2	59	1	5	222	ST-206	Generalist	[2]	Susceptible
TW16644	PRJNA305291	SAMN04323593	2012	Yes	Urban	1735199	1.7	30.4%	96	29.5	23.3	95.2%	1786	IF	2	1	1	5	22	1	8	454	ST-21	Generalist	[2]	Susceptible
TW16645	PRJNA305291	SAMN04323594	2012			1657056	1.7	30.8%	364	7.9	4.4	86.5%	1724	IC	9	2	4	62	4	5	17	929	ST-257	Chicken	[2]	TET
TW16646	PRJNA305291	SAMN04323595	2012		Rural	1686417	1.7	30.6%	146	21.6	11.2	96.7%	1720	IF	2	1	2	3	2	1	5	982	ST-21	Cattle	[1]	TET
TW16647	PRJNA305291	SAMN04323596	2012	Yes	Rural	1674944	1.7	30.6%	142	20.6	10.2	96.5%	1698	IF	2	1	2	3	2	1	5	982	ST-21	Cattle	[1]	TET
TW16648	PRJNA305291	SAMN04323597	2012	No	Urban	1707023	1.7	30.6%	286	10.6	5.6	90.7%	1758	ID	7	17	5	2	10	3	6	353	ST-353	Chicken	[1]	TET
TW16649	PRJNA305291	SAMN04323598	2012	Yes	Rural	1625416	1.6	30.5%	66	50.2	30.1	93.2%	1651	IB	1	1	2	83	2	3	6	922				Susceptible
TW16651	PRJNA305291	SAMN04323599	2012	No	Rural	1664190	1.7	30.6%	73	47.7	30.1	89.0%	1700	II	4	7	10	4	10	7	1	2109	ST-45	Generalist	[2]	Susceptible
TW16655	PRJNA305291	SAMN04323600	2012		Urban	1636957	1.6	30.9%	482	5.6	3.1	85.4%	1630	ID	8	2	2	212	309	253	147	2083				CIPTET
TW16656	PRJNA305291	SAMN04323601	2012	No	Rural	1722390	1.7	30.3%	174	23.2	10.7	89.8%	1799	IA	2	29	4	48	10	25	57	2875				Susceptible
TW16657	PRJNA305291	SAMN04323602	2012	Yes	Urban	1807972	1.8	30.6%	56	93.6	48.9	88.7%	1886	II	296	34	27	33	45	36	329	5221				CIPAZIERYCLITEL
TW16662	PRJNA305291	SAMN04323603	2012	Yes	Urban	1666881	1.7	30.6%	49	81.1	38.5	88.9%	1715	III	1	2	3	4	5	9	3	42	ST-42	Cattle	[2,3]	TET
TW16663	PRJNA305291	SAMN04323604	2012	No	Rural	1697791	1.7	30.7%	268	12.5	6.2	88.6%	1730	IC	9	2	4	62	4	5	17	929	ST-257	Chicken	[2]	TET
TW16665	PRJNA305291	SAMN04323605	2012	No	Rural	1687672	1.7	30.6%	228	13	7.2	88.7%	1740	IC	9	2	4	62	4	5	17	929	ST-257	Chicken	[2]	TET
TW16670	PRJNA305291	SAMN04323606	2012		Rural	1642571	1.6	30.5%	38	99	70.8	94.8%	1670	IF	2	4	1	2	7	1	5	48	ST-48	Generalist	[2]	Susceptible
TW16676	PRJNA305291	SAMN04323607	2012		Urban	1499248	1.5	30.9%	269	9.2	5.2	85.4%	1485	IA	2	1	4	28	58	29	58	6788	ST-1332			TET
TW16689	PRJNA305291	SAMN04323608	2012	No	Urban	1629125	1.6	31.0%	323	8.8	5.1	87.6%	1728	III	91	2	42	4	169	9	8	986				AZICIP
TW16693	PRJNA305291	SAMN04323609	2012	Yes	Rural	1697407	1.7	30.5%	58	75.6	44.5	97.8%	1755	IF	2	1	2	3	2	1	5	982	ST-21	Cattle	[1]	TET
TW16694	PRJNA305291	SAMN04323610	2012	No	Urban	1735820	1.7	30.3%	26	129.4	102.5	97.9%	1793	IF	2	1	2	3	2	1	5	982	ST-21	Cattle	[1]	TET
TW16695	PRJNA305291	SAMN04323611	2012	No	Urban	1621496	1.6	30.6%	95	37.1	21.5	95.6%	1645	IF	2	1	12	462	2	1	5	5727	ST-21	Generalist	[2]	CIP
TW16698	PRJNA305291	SAMN04323612	2012	No	Rural	1776100	1.8	30.4%	218	18.8	8.5	93.1%	1834	ID	24	2	2	2	10	3	1	464	ST-464	Chicken	[1]	CIPTET
TW16702	PRJNA305291	SAMN04323613	2012	Yes	Urban	1763389	1.8	30.2%	86	103.8	77.4	92.1%	1829	ID	7	2	5	2	156	3	6	939	ST-353	Chicken	[2]	Susceptible
TW16706	PRJNA305291	SAMN04323614	2012	No	Rural	1734426	1.7	30.3%	53	102.6	48.7	93.6%	1795	ID	24	2	2	2	10	3	1	464	ST-464	Chicken	[1]	CIPTET
TW16707	PRJNA305291	SAMN04323615	2012			1677206	1.7	30.4%	53	83.6	45.9	93.6%	1696	IB	1	1	2	83	2	3	6	922				TET
TW16709	PRJNA305291	SAMN04323616	2012		Urban	1783558	1.8	30.3%	83	77.5	49.6	93.8%	1855	ID	7	17	5	2	10	3	6	353	ST-353	Chicken	[1]	TET
TW16719	PRJNA305291	SAMN04323617	2012		Urban	1731114	1.7	30.3%	39	102.3	50.4	93.7%	1804	ID	7	17	5	2	10	3	6	353	ST-353	Chicken	[1]	Susceptible
TW16720	PRJNA305291	SAMN04323618	2012	Yes	Rural	1665431	1.7	30.4%	40	81.1	59.3	97.8%	1709	IF	2	1	1	3	2	1	5	21	ST-21	Generalist	[1,2]	Susceptible
TW16723	PRJNA951423	SAMN34041911	2012	Yes	Urban	1651060	1.7	30.5%	50	73.9	45.2	97.4%	1704	IF	2	1	1	3	2	1	5	21	ST-21	Generalist	[1,2]	Susceptible
TW16724	PRJNA305291	SAMN04323620	2012			1649597	1.6	31.0%	205	14	8.1	91.4%	1661	IB	3	1	5	84	11	11	6	467	ST-49			TET
TW16725	PRJNA305291	SAMN04323621	2012	Yes	Urban	1628226	1.6	30.7%	171	17.2	10.7	89.2%	1667	II	4	7	10	4	1	7	1	45	ST-45	Chicken	[1]	TET
TW16727	PRJNA305291	SAMN04323622	2012	Yes	Rural	1695332	1.7	30.8%	365	8.1	4.4	88.2%	1739	ID	7	17	5	2	13	3	6	3510	ST-353	Chicken	[2]	CIPTET
TW16728	PRJNA305291	SAMN04323623	2012	Yes	Urban	1642945	1.6	30.5%	43	89.6	63	94.7%	1672	IF	2	4	1	2	7	1	5	48	ST-48	Generalist	[2]	Susceptible
TW16733	PRJNA305291	SAMN04323624	2012			1708661	1.7	30.5%	53	57.4	31.1	95.7%	1765	IF	2	1	12	3	2	1	5	50	ST-21	Chicken	[1]	TET
TW16735	PRJNA305291	SAMN04323625	2012	No	Urban	1839015	1.8	30.9%	264	11.1	6.3	85.5%	1919	II	296	34	27	33	697	545	213	7010				CIPTET
TW16892	PRJNA368990	SAMN11785417	2013		Urban	1751920	1.8	30.3%	17	213.2	104.8	96.3%	1817	IF	2	1	12	3	2	1	5	50	ST-21	Chicken	[1]	TET
TW19100	PRJNA368990	SAMN10506160	2013	No	Rural	1617078	1.6	30.4%	37	91.3	41.9	92.5%	1656	IF	1	1	2	2	225	3	17	1244	ST-61	Cattle	[2,3]	TET
TW19101	PRJNA951423	SAMN34041912	2013	No	Urban	1712859	1.7	30.4%	20	199.2	114.9	96.0%	1777	IF	1	1	2	3	2	1	5	10579				CIPTETCLI
TW19102	PRJNA951423	SAMN34041913	2013			1671317	1.7	30.5%	19	184.7	130.3	98.0%	1725	IF	2	1	2	3	2	1	5	982	ST-21	Cattle	[1]	CIPTET
TW19103	PRJNA951423	SAMN34041914	2013		Urban	1878539	1.9	30.1%	54	123.3	84	93.8%	1997	ID	8	2	5	53	11	3	105	1212	ST-607	Chicken	[1]	TET
TW19104	PRJNA951423	SAMN34041915	2013	Yes	Urban	1614861	1.6	30.5%	24	205.1	153.7	90.3%	1627	II	4	7	10	4	1	7	1	45	ST-45	Chicken	[1]	GENTET
TW19105	PRJNA951423	SAMN34041916	2013	No	Rural	1729189	1.7	30.3%	44	209.2	131.7	93.5%	1796	ID	24	2	2	2	10	3	1	464	ST-464	Chicken	[1]	Susceptible
TW19107	PRJNA951423	SAMN34041917	2013			1714048	1.7	30.2%	39	107.3	93.9	93.5%	1763	ID	188	313	5	2	89	5	6	5326				Susceptible
TW19108	PRJNA368990	SAMN10506171	2013			1780576	1.8	30.3%	47	105.7	49.7	94.1%	1832	IE	7	84	5	10	11	3	6	1036	ST-353	Chicken	[2]	CIPTET
TW19109	PRJNA951423	SAMN34041918	2013	No	Urban	1833287	1.8	30.0%	86	165.1	116.5	87.8%	1897	II	27	33	22	49	43	9	31	1223	ST-1275			Susceptible
TW19110	PRJNA951423	SAMN34041919	2013	Yes	Rural	1844934	1.8	30.1%	45	166.7	104.1	93.3%	1948	ID	8	2	5	53	11	3	1	607	ST-607	Chicken	[1]	CIPTET
TW19111	PRJNA951423	SAMN34041920	2013	Yes	Urban	1781774	1.8	30.2%	20	184	113.9	98.1%	1849	IF	2	1	2	3	2	1	5	982	ST-21	Cattle	[1]	Susceptible
TW19112	PRJNA951423	SAMN34041921	2013	Yes	Urban	1596427	1.6	30.4%	21	184.2	139.6	87.6%	1604	II	4	7	40	4	42	51	1	267	ST-283	Generalist	[1]	Susceptible
TW19113	PRJNA951423	SAMN34041922	2011	Yes	Rural	1801820	1.8	30.3%	102	58	25.4	92.2%	1879	ID	7	2	5	2	2	3	6	2132	ST-353	Chicken	[2]	CIP
TW19114	PRJNA951423	SAMN34041923	2013			1625729	1.6	30.5%	18	188.3	106.6	94.3%	1668	IF	7	4	5	2	11	1	5	429	ST-48	Generalist	[2]	Susceptible
TW19115	PRJNA951423	SAMN34041924	2013	Yes	Urban	1604243	1.6	30.5%	22	203.6	135.4	90.3%	1615	II	4	7	10	4	1	7	1	45	ST-45	Chicken	[1]	TET
TW19116	PRJNA951423	SAMN34041925	2013			1633687	1.6	30.4%	18	234.5	144.9	88.5%	1662	II	4	7	10	4	42	7	1	137	ST-45	Chicken	[1]	Susceptible
TW19117	PRJNA951423	SAMN34041926	2013	Yes	Urban	1632762	1.6	30.5%	31	207.2	185.2	89.6%	1633	II	4	7	10	4	1	7	1	45	ST-45	Chicken	[1]	TET
TW19118	PRJNA951423	SAMN34041927	2013	No	Rural	1767340	1.8	30.3%	32	188.3	148.5	93.1%	1833	ID	8	17	5	2	10	59	6	400	ST-353	Chicken	[1,2]	CIPTET
TW19119	PRJNA951423	SAMN34041928	2013	No	Rural	1782083	1.8	30.3%	39	139.7	102.2	92.7%	1865	ID	9	2	1	10	22	3	6	2275	ST-52	Generalist	[1]	TET
TW19120	PRJNA951423	SAMN34041929	2013		Urban	1614544	1.6	30.5%	25	126.7	89.5	93.1%	1654	ID	2	17	5	10	11	3	6	8741	ST-353	Chicken	[1,2]	CIP
TW19122	PRJNA368990	SAMN10506167	2013	Yes	Rural	1638834	1.6	30.5%	33	151.5	52	92.7%	1701	ID	9	25	2	10	22	3	6	52	ST-52	Generalist	[1]	Susceptible
TW19123	PRJNA368990	SAMN11785415	2013	Yes	Urban	1681818	1.7	30.3%	35	181.7	107.2	92.4%	1724	ID	7	17	5	2	10	3	6	353	ST-353	Chicken	[1]	Susceptible
TW19124	PRJNA951423	SAMN34041930	2013	Yes	Urban	1649770	1.6	30.4%	20	176.9	126.8	90.8%	1725	IC	9	2	4	62	4	5	17	929	ST-257	Chicken	[2]	CIPTET
TW19126	PRJNA368990	SAMN10506055	2013	No	Rural	1665494	1.7	30.5%	85	33.5	18.3	97.6%	1716	IF	2	1	2	3	2	1	5	982	ST-21	Cattle	[1]	Susceptible
TW19128	PRJNA368990	SAMN10506188	2013	No	Rural	1758020	1.8	30.3%	83	43.3	28.3	93.8%	1837	ID	9	2	2	2	11	5	6	824	ST-257	Chicken	[2]	CIPTET
TW19129	PRJNA951423	SAMN34041931	2013	Yes	Urban	1718821	1.7	30.3%	38	128.8	90.5	94.9%	1787	IF	2	21	5	2	59	1	5	222	ST-206	Generalist	[2]	Susceptible

TW19130	PRJNA951423	SAMN34041979	2013			1658911	1.7	30.4%	13	293.7	194.9	91.1%	1726	IC	9	2	4	62	4	5	17	929	ST-257	Chicken	[2]	TET
TW19131	PRJNA951423	SAMN34041980	2013		Rural	1700025	1.7	30.4%	33	132.2	100.9	96.2%	1761	IF	2	1	12	3	2	1	5	50	ST-21	Chicken	[1]	Susceptible
TW19132	PRJNA951423	SAMN34041981	2013	No	Rural	1813417	1.8	30.1%	36	175.3	103.7	93.0%	1903	ID	8	2	5	831	11	3	1	12343¥				TET
TW19133	PRJNA951423	SAMN34041982	2012	Yes	Rural	1704349	1.7	30.7%	233	16.5	7	90.8%	1770	ID	24	2	2	2	10	3	1	464	ST-464	Chicken	[1]	Susceptible
TW19134	PRJNA951423	SAMN34041983	2013	No	Rural	1774109	1.8	30.3%	25	154.7	102	95.1%	1841	IF	2	4	1	24	7	1	5	6639	ST-48	Generalist	[2]	TET
TW19135	PRJNA951423	SAMN34041984	2013	Yes	Rural	1739814	1.7	30.3%	33	141	71.9	94.0%	1839	IE	7	84	5	10	119	178	26	1911				AZICLI
TW19136	PRJNA951423	SAMN34041985	2011	Yes	Urban	1431867	1.4	31.9%	668	3	1.7	77.2%	1533	ID	7	17	5	2	10	3	6	353	ST-353	Chicken	[1]	Susceptible
TW19137	PRJNA951423	SAMN34041986	2013	Yes	Urban	1684794	1.7	30.4%	15	197.6	120.6	94.0%	1704	IB	1	1	2	83	2	3	6	922				TET
TW19138	PRJNA951423	SAMN34041987	2013	Yes	Rural	1676557	1.7	30.4%	30	154.3	111.7	93.7%	1698	IB	1	1	2	83	2	3	6	922				TET
TW19139	PRJNA951423	SAMN34041988	2013		Urban	1639809	1.6	30.5%	14	196.6	153.8	94.9%	1672	IF	2	4	1	2	7	1	5	48	ST-48	Generalist	[2]	Susceptible
TW19140	PRJNA368990	SAMN10506066	2013		Urban	1740585	1.7	30.4%	74	49.7	27.8	93.8%	1806	IE	7	4	5	2	11	3	165	1838	ST-353	Chicken	[2]	Susceptible
TW19142	PRJNA951423	SAMN34041989	2013			1733902	1.7	30.3%	49	132.7	85.1	92.9%	1800	ID	7	17	5	2	10	3	6	353	ST-353	Chicken	[1]	Susceptible
TW19144	PRJNA368990	SAMN10506069	2013	Yes	Urban	1826559	1.8	30.3%	63	64.9	29.3	95.1%	1918	IF	62	4	5	2	2	1	5	572	ST-206	Generalist	[2]	CIPTET
TW19145	PRJNA951423	SAMN34041990	2013	No	Urban	1682419	1.7	30.3%	26	186.2	104.9	94.0%	1724	IE	7	4	5	2	11	3	165	1838	ST-353	Chicken	[2]	Susceptible
TW19147	PRJNA951423	SAMN34041991	2013	No	Rural	1683626	1.7	30.4%	20	131.9	103	95.0%	1728	IF	6	4	5	2	2	1	5	122	ST-206	Generalist	[2]	TET
TW19149	PRJNA368990	SAMN10506129	2014		Urban	1694443	1.7	30.3%	44	89.5	41.9	92.4%	1759	IF	1	1	2	2	225	3	17	1244	ST-61	Cattle	[2,3]	TET
TW19151	PRJNA368990	SAMN10506132	2014	No	Urban	1762257	1.8	30.2%	76	62.6	27.7	92.7%	1839	ID	8	2	5	10	11	3	1	10349				CIPTET
TW19152	PRJNA368990	SAMN11784736	2014	Yes	Urban	1686547	1.7	30.4%	17	176	103.6	92.3%	1756	IF	1	4	2	2	6	3	17	61	ST-61	Cattle	[2,3]	TET
TW19153	PRJNA368990	SAMN11845845	2014	Yes	Rural	1719640	1.7	30.1%	85	57	30	90.4%	1774	IA	37	4	4	48	13	25	57	1030				Susceptible
TW19154	PRJNA951423	SAMN34041992	2014	No	Rural	1792431	1.8	30.2%	31	191.1	104.5	92.6%	1886	ID	14	2	6	15	914	3	6	9138				CIPTET
TW19237	PRJNA951423	SAMN34041993	2013	Yes	Urban	1729189	1.7	30.3%	44	209.2	131.7	93.5%	1796	ID	7	17	5	2	10	3	6	353	ST-353	Chicken	[1]	TET
TW19238	PRJNA951423	SAMN34041994	2013	Yes	Urban	1699942	1.7	30.2%	34	248.5	126.6	93.5%	1744	ID	7	17	5	2	10	3	6	353	ST-353	Chicken	[1]	TET
TW19239	PRJNA951423	SAMN34041995	2014		Urban	1661998	1.7	30.4%	16	154.4	104.4	98.1%	1704	IF	2	1	1	3	2	1	5	21	ST-21	Generalist	[1,2]	Susceptible
TW19240	PRJNA951423	SAMN34041996	2014	No	Urban	1747717	1.7	30.2%	36	140.8	103.8	93.2%	1791	ID	7	17	5	2	10	3	1	9062	ST-353	Chicken	[2]	CIPTETTEL
TW19241	PRJNA951423	SAMN34041997	2014	Yes	Urban	1813675	1.8	30.2%	23	230.4	130.9	90.0%	1889	III	1	3	6	4	3	3	3	22	ST-22	Cattle	[1]	TET
TW19242	PRJNA368990	SAMN11785419	2014	No	Rural	1670360	1.7	30.5%	24	115.7	103.6	97.4%	1733	IF	2	76	2	3	2	1	5	597	ST-21	Generalist	[2]	Susceptible
TW19243	PRJNA951423	SAMN34041998	2014	Yes	Urban	1681109	1.7	30.3%	86	49.6	23.6	93.5%	1729	ID	7	2	5	10	10	37	129	1698				TET
TW19244	PRJNA951423	SAMN34041999	2014		Urban	1889890	1.9	30.4%	32	186.2	110.2	88.0%	2008	II	296	34	27	33	697	545	213	7010				CIPTET
TW19246	PRJNA951423	SAMN34042000	2014	Yes	Urban	1596158	1.6	30.4%	19	184.2	145.3	87.5%	1603	II	4	7	40	4	42	51	1	267	ST-283	Generalist	[1]	Susceptible
TW19248	PRJNA951423	SAMN34042001	2014	No	Urban	1640185	1.6	30.4%	13	296.8	179.9	91.9%	1689	ID	8	10	2	2	11	12	6	354	ST-354	Chicken	[1,2]	TET
TW19249	PRJNA951423	SAMN34042002	2014	Yes	Urban	1714673	1.7	30.3%	98	41.2	19.2	93.4%	1778	ID	7	17	5	2	10	3	6	353	ST-353	Chicken	[1]	Susceptible
TW19251	PRJNA951423	SAMN34042003	2014	Yes	Urban	1743495	1.7	30.2%	32	160.9	106.3	91.4%	1799	II	10	25	16	19	10	5	7	6674	ST-403	Cattle	[1]	Susceptible
TW19252	PRJNA368990	SAMN10506076	2014			1793727	1.8	30.2%	56	81.2	42.5	94.1%	1883	ID	9	2	2	2	11	5	6	824	ST-257	Chicken	[2]	CIPTET
TW19253	PRJNA951423	SAMN34042004	2014	Yes	Rural	1788602	1.8	30.3%	27	164.1	121	98.0%	1880	IF	2	1	2	3	2	1	5	982	ST-21	Cattle	[1]	TET
TW19254	PRJNA951423	SAMN34042005	2014		Urban	1733460	1.7	30.3%	24	181.1	119.6	93.9%	1799	ID	8	17	5	2	10	3	1	524	ST-353	Chicken	[1,2]	CIPTET
TW19255	PRJNA951423	SAMN34042006	2014		Urban	1714983	1.7	30.4%	20	218.4	138.5	95.4%	1777	IF	2	1	1	3	140	3	5	806	ST-21	Cattle	[1]	TET
TW19256	PRJNA951423	SAMN34042007	2014		Urban	1726947	1.7	30.3%	37	148.5	118.5	92.4%	1775	ID	7	17	5	2	10	3	6	353	ST-353	Chicken	[1]	TET
TW19257	PRJNA951423	SAMN34042008	2014	Yes	Rural	1740219	1.7	30.4%	13	332.5	184.7	95.6%	1818	IF	2	1	1	83	40	3	5	12344¥				Susceptible
TW19258	PRJNA368990	SAMN11845848	2014	No	Rural	1641550	1.6	30.5%	73	48.6	23.7	94.7%	1669	IF	2	4	1	2	7	1	5	48	ST-48	Generalist	[2]	Susceptible
TW19259	PRJNA368990	SAMN10506078	2014	Yes	Urban	1681109	1.7	30.3%	86	49.6	23.6	93.5%	1729	ID	24	2	2	2	10	3	1	464	ST-464	Chicken	[1]	CIP
TW19262	PRJNA951423	SAMN34042009	2014	No	Rural	1666354	1.7	30.4%	24	154.1	103.6	97.5%	1700	IF	2	17	2	3	2	1	5	883	ST-21	Generalist	[2]	CIPTET
TW19263	PRJNA951423	SAMN34042010	2014	Yes	Urban	1692246	1.7	30.4%	27	182.8	90.2	90.7%	1727	II	1	6	60	24	12	28	1	508	ST-508			AZI
TW19265	PRJNA951423	SAMN34042011	2014	Yes	Rural	1637024	1.6	30.5%	30	127.8	63	92.8%	1693	ID	7	2	5	2	2	3	6	2132	ST-353	Chicken	[2]	CIPTET
TW19266	PRJNA368990	SAMN10506165	2014	Yes	Urban	1773899	1.8	30.3%	57	95.2	47.1	93.2%	1841	ID	7	17	2	2	10	3	6	3515	ST-353	Chicken	[2]	TET
TW19267	PRJNA368990	SAMN11845849	2014	No	Urban	1677377	1.7	30.3%	48	79.3	43.1	93.0%	1719	IF	2	4	119	25	11	3	5	1039				Susceptible
TW19268	PRJNA951423	SAMN34042012	2014	No	Urban	1641984	1.6	30.4%	46	100.8	69.6	95.3%	1668	IF	2	1	12	3	2	1	5	50	ST-21	Chicken	[1]	Susceptible
TW19269	PRJNA368990	SAMN10506148	2014	No	Rural	1827667	1.8	30.1%	83	182.1	65	92.8%	1895	ID	7	2	5	2	11	3	6	2517	ST-353	Chicken	[2]	TET
TW19270	PRJNA368990	SAMN10506138	2014	No	Rural	1820577	1.8	30.2%	96	51	24.6	93.7%	1916	ID	9	2	5	2	11	3	1	1707	ST-607	Chicken	[1]	CIPTET
TW19271	PRJNA368990	SAMN10506141	2014	No	Urban	1679075	1.7	30.5%	83	33.4	20.2	94.6%	1742	IF	2	1	2	3	2	1	5	982	ST-21	Cattle	[1]	TET
TW19272	PRJNA368990	SAMN10506152	2014	Yes	Urban	1714673	1.7	30.3%	98	41.2	19.2	93.4%	1778	ID	24	2	2	2	10	3	1	464	ST-464	Chicken	[1]	CIP
TW19273	PRJNA951423	SAMN34042013	2014	No	Urban	1655486	1.7	30.4%	16	180.3	157.2	92.6%	1725	IB	3	1	5	84	11	11	6	467	ST-49			Susceptible
TW19274	PRJNA368990	SAMN11845846	2014	Yes	Rural	1710293	1.7	30.4%	38	178	88.9	97.2%	1764	IF	2	1	2	3	2	1	5	982	ST-21	Cattle	[1]	CIPTET
TW19276	PRJNA368990	SAMN10734826	2014	No	Urban	1662486	1.7	30.5%	96	31.8	17.6	94.8%	1710	IF	2	1	1	3	140	3	5	806	ST-21	Cattle	[1]	Susceptible
TW19277	PRJNA368990	SAMN10506159	2014	No	Rural	1731457	1.7	30.3%	100	34.4	19.2	93.4%	1798	ID	24	2	2	2	10	3	1	464	ST-464	Chicken	[1]	CIP
TW19278	PRJNA368990	SAMN10506174	2014		Rural	1688791	1.7	30.4%	78	42.2	23.3	92.2%	1750	IF	1	1	2	2	225	3	17	1244	ST-61	Cattle	[2,3]	TET
TW19279	PRJNA368990	SAMN10506175	2014	Yes	Rural	1677338	1.7	30.4%	63	65.9	55.3	95.1%	1724	IF	2	21	5	2	59	1	5	222	ST-206	Generalist	[2]	Susceptible
TW19281	PRJNA368990	SAMN10506176	2014	Yes	Urban	1708107	1.7	30.4%	29	154.7	87.1	94.9%	1775	IF	2	21	5	2	59	1	5	222	ST-206	Generalist	[2]	Susceptible
TW19282	PRJNA951423	SAMN34042014	2014	Yes	Rural	1618323	1.6	30.5%	15	295.5	154.5	95.8%	1657	IF	2	1	1	3	2	1	6	8	ST-21	Cattle	[1]	TET
TW19283	PRJNA951423	SAMN34042015	2011	Yes	Urban	1598049	1.6	31.5%	692	3.4	1.9	81.9%	1638	ID	7	2	5	10	10	37	129	1698				TET
TW19284	PRJNA951423	SAMN34042016	2014	Yes	Urban	1680905	1.7	30.4%	17	161.4	104.3	95.2%	1726	IF	2	4	1	4	2	62	5	2782	ST-21	Generalist	[2]	Susceptible
TW19285	PRJNA951423	SAMN34042017	2014	No	Rural	1640144	1.6	30.5%	18	161.1	104.3	95.0%	1666	IF	2	4	1	4	19	1	5	918	ST-48	Generalist	[2]	Susceptible
TW19288	PRJNA368990	SAMN10506073	2014	No	Urban	1664186	1.7	30.5%	100	27.7	18	95.9%	1712	IF	2	1	12	3	2	1	5	50	ST-21	Chicken	[1]	Susceptible
TW19290	PRJNA368990	SAMN10506110	2014		Rural	1626950	1.6	30.4%	61	60	26.2	86.6%	1692	II	10	2	50	62	91	73	45	468				Susceptible
TW19292	PRJNA368990	SAMN10506065	2014	Yes	Urban	1774852	1.8	30.2%	48	149.2	66.1	93.6%	1866	ID	7	2	5	10	10	37	129	1698				CIPTET
TW19293	PRJNA368990	SAMN10506077	2014	No	Rural	1698705	1.7	30.4%	51	86.9	47.6	97.6%	1743	IF	2	1	1	3	2	1	5	21	ST-21	Generalist	[1,2]	CIP
TW19294	PRJNA368990	SAMN10506067	2014		Urban	1640177	1.6	30.5%	51	64.2	40.9	94.9%	1673	IF	2	4	1	4	19	62	5	475	ST-48	Generalist	[2]	CIP
TW19295	PRJNA368990	SAMN10506136	2014		Urban	1720218	1.7	30.4%	63	70	42.5	96.1%	1778	IF	2	1	12	3	2	1	5	50	ST-21	Chicken	[1]	Susceptible
TW19296	PRJNA368990	SAMN10506137	2014	Yes	Urban	1784982	1.8	30.4%	54	154.7	62.8	96.3%	1860	IF	2	1	12	3	2	1	5	50	ST-21	Chicken	[1]	TET
TW19297	PRJNA368990	SAMN10506162	2014	No	Urban	1644395	1.6	30.5%	28	117.1	68.7	95.2%	1675	IF	150	4	1	2	7	1	5	2521	ST-48	Generalist	[2]	Susceptible
TW19298	PRJNA368990	SAMN10506172	2014	Yes	Urban	1771863	1.8	30.3%	60	148.1	94.2	92.8%	1853	ID	7	17	5	2	10	3	6	353	ST-353	Chicken	[1]	Susceptible
TW19299	PRJNA368990	SAMN10506168	2014	Yes	Urban	1778539	1.8	30.1%	34	177.8	106.1	91.2%	1836	II	10	27	16	19	10	5	7	403	ST-403	Cattle	[1]	CIPTET
TW19300	PRJNA368990	SAMN10506133	2014	No	Rural	1641438	1.6	30.4%	92	30.6	19.2	90.4%	1672	II	4	7	10	4	1	7	1	45	ST-45	Chicken	[1]	Susceptible
TW19301	PRJNA368990	SAMN10506144	2014			1696806	1.7	30.3%	129	24.1	13.1	93.2%	1741	ID	7	17	5	2	13	3	6	3510	ST-353	Chicken	[2]	TET
TW19302	PRJNA368990	SAMN10506147	2014			1681552	1.7	30.3%	76	42.2	24.9	93.5%	1733	ID	7	17	5	2	10	3	6	353	ST-353	Chicken	[1]	Susceptible
TW19303	PRJNA368990	SAMN10506156	2014	Yes	Urban	1765940	1.8	30.2%	78	58.9	30.4	92.9%	1844	ID	8	2	5	53	11	3	1	607	ST-607	Chicken	[1]	Susceptible
TW19306	PRJNA368990	SAMN10506181	2014		Urban	1632528	1.6	30.4%	67	62.5	40.2	92.8%	1685	ID	7	17	2	15	23	3	12	51	ST-443	Chicken	[2]	TET
TW19307	PRJNA368990	SAMN10506184	2014		Urban	1748674	1.7	30.4%	73	45.1	21.6	96.1%	1816	IF	2	1	12	3	2	1	5	50	ST-21	Chicken	[1]	TET
TW19308	PRJNA368990	SAMN10506186	2014			1722124	1.7	30.3%	66	87.1	50.3	92.5%	1785	ID	9	17	2	2	11	12	356	5837	ST-354	Chicken	[2]	TET
TW19309	PRJNA368990	SAMN10506059	2014			1627184	1.6	30.4%	32	154.5	84.9	92.7%	1681	ID	7	17	12	15	23	3	12	1904	ST-443	Chicken	[2]	Susceptible
TW19310	PRJNA368990	SAMN10506063	2014	No	Urban	1662967	1.7	30.4%	25	133	89.7	93.2%	1724	IF	7	4	1	68	11	68	6	7231				CIPTET
TW19311	PRJNA368990	SAMN10506071	2014	Yes	Urban	1637024	1.6	30.5%	30	127.8	63.0	92.8%	1693	ID	9	25	2	10	22	3	6	52	ST-52	Generalist	[1]	Susceptible
TW19312	PRJNA368990	SAMN10506074	2014	No	Rural	1625311	1.6	30.5%	33	176	136.1	89.4%	1621	II	4	7	10	4	1	7	1	45	ST-45	Chicken	[1]	CIPTET
TW19313	PRJNA368990	SAMN10506111	2014	Yes	Urban	1704904	1.7	30.3%	23	176.9	112.4	91.0%	1774	IC	9	2	4	62	4	5	17	929	ST-257	Chicken	[2]	TET
TW19314	PRJNA368990	SAMN10506178	2014	Yes	Urban	1635649	1.6	30.5%	31	151.5	72.2	92.7%	1696	ID	9	25	2	10	22	3	6	52	ST-52	Generalist	[1]	Susceptible
TW19315	PRJNA368990	SAMN10506185	2014			1637691	1.6	30.5%	23	151.5	117.1	92.8%	1694	ID	9	25	2	10	22	3	6	52	ST-52	Generalist	[1]	Susceptible
TW19316	PRJNA368990	SAMN10506057	2014			1802231	1.8	30.2%	151	23.5	12.8	93.8%	1884	ID	9	2	2	2	11	5	6	824	ST-257	Chicken	[2]	CIPTET
TW19317	PRJNA368990	SAMN10506068	2014			1699083	1.7	30.4%	75	48.6	25.7	90.8%	1763	IC	9	2	4	62	4	5	17	929	ST-257	Chicken	[2]	TET
TW19318	PRJNA368990	SAMN10506070	2014		Urban	1698135	1.7	30.4%	70	49	31.8	90.8%	1759	IC	9	2	4	62	4	5	17	929	ST-257	Chicken	[2]	TET
TW19319	PRJNA368990	SAMN10506177	2014			1634206	1.6	30.5%	62	49.9	30.5	92.6%	1695	ID	9	25	2	10	22	3	6	52	ST-52	Generalist	[1]	Susceptible
TW19320	PRJNA368990	SAMN10506154	2014		Rural	1680479	1.7	30.4%	43	86.8	53.2	93.7%	1703	IB	1	1	2	83	2	3	6	922				TET
TW19321	PRJNA368990	SAMN10506163	2014	No	Urban	1592195	1.6	30.4%	21	155.3	116.2	89.6%	1634	IA	2	61	4	64	74	25	23	1034	ST-1034			Susceptible
TW19322	PRJNA368990	SAMN10506164	2014	Yes	Rural	1681722	1.7	30.4%	81	50.8	24.7	93.7%	1698	IB	1	1	2	83	2	3	6	922				TET
TW19323	PRJNA368990	SAMN10506166	2014	Yes	Rural	1645001	1.6	30.5%	37	103.1	46.1	94.9%	1674	IF	2	4	1	2	7	1	5	48	ST-48	Generalist	[2]	Susceptible

* Recovered from a patient residing outside of Michigan

† "Yes" refers to strains from cases who did not report recent travel outside of Michigan; blank spaces indicate missing data.

**Clusters were defined for strains that grouped together with > 80% bootstrap support in the 615 core-gene RaXML phylogeny

‡ Antibiotic resistance abbreviations: CIP, ciprofloxacin; TET, tetracycline; AZI, azithromycin; CLI, clarithromycin; TEL, telithromycin; ERY, erythromycin; FFN, phenicol; GEN, gentamicin

¥ New defined Sequence type

[1] Cobo-Diaz et al. Front Microbiol. 2021;12:662144

[2] Sheppard et al. Mol Ecol. 2014; 23(10): 2442–2451.

Table S2. Association between resistance to ciprofloxacin (CIP) or tetracycline (TET) and sequence cluster designation as determined by the core rooted gene phylogeny for 214 *Campylobacter jejuni* strains.

	Any CIP resistance (n=48)				Any TET resistance (n=120)			
**Clusters**	**No.**	**(%)[a]**	**OR (95% CI)[b]**	***p* value[c]**	**No.**	**(%)[a]**	**OR (95% CI)[b]**	***p* value[c]**
Subcluster IA (n = 5)	0	(0.0%)	---	--	1	(0.8%)	---	0.2
Subcluster IB (n = 10)	0	(0.0%)	---	---	7	(5.8%)	---	0.5
Subcluster IC (n = 9)	1	(2.1%)	---	0.7	9	(7.5%)	----	0.005
Subcluster ID (n = 70)	23	(47.9%)	2.3 (1.20-4.50)	0.01	41	(34.2%)	1.2 (0.65-2.07)	0.7
Subcluster IE (n = 6)	1	(2.1%)	----	1.0	3	(2.5%)	---	1.0
Subcluster IF (n = 78)	16	(33.3%)	0.8 (0.43-1.65)	0.7	44	(36.7%)	1.0 (0.58-1.79)	1.0
Cluster II (n = 31)	6	(12.5%)	0.8 (0.31-2.09)	0.8	12	(10.0%)	0.4 (0.20-0.96)	0.06
Cluster III (n = 5)	1	(2.1%)	----	1.0	3	(2.5%)	---	1.0

[a] Percentages were calculated using the total number of resistant isolates as the denominator for both groups.
[b] The odds ratio (OR) and 95% confidence interval (CI) is presented and were calculated separately for any CIP and any TET resistance relative to all other strains.
[c] The Fisher's Exact Test was used for variables with ≤5 in one cell and no ORs could be calculated.

Table S3: Univariate analysis to identify factors associated with pangenomic sequence clusters defined based on 615 core genes among 214 *Campylobacter jejuni* isolates from Michigan, 2011-2014

	Cluster I (n=178)		Cluster II (n=36)		
**Characteristics** [a]	**No.**	**(%)**	**No. (%)**	**OR (95% CI)** [b]	**p-value** [c]
Age (years)					
0-9 (n=66)	58	(32.8)	8 (22.2)	2.8 (1.08-7.05)	0.03
10-18 (n=17)	14	(7.9)	3 (8.3)	---	0.53
19-40 (n=54)	46	(26.0)	8 (22.2)	1.0	---
41-65 (n=58)	42	(23.7)	16 (44.4)	2.2 (0.85-5.64)	0.10
≥65 (n=18)	17	(9.6)	1 (2.8)	---	0.06
Sex					
Male (n=113)	92	(53.8)	21 (60.0)	1.0	
Female (n=93)	79	(46.2)	14 (40.0)	1.3 (0.62-2.70)	0.50
Self-reported race [d]					
White/Caucasian (n=136)	113	(81.3)	23 (69.7)	1.0	
Non-White/Other (n=36)	26	(18.7)	10 (30.3)	0.5 (0.22-1.25)	0.14
Season					
Winter and spring (n=54)	45	(25.3)	9 (25.0)	1.0	
Summer and fall (n=160)	133	(74.7)	27 (75.0)	1.0 (0.43-2.25)	1.0
Travel in the past month					
None (n=87)	68	(55.3)	19 (76.0)	1.0	
Domestic (n=35)	30	(25.4)	5 (20.0)	1.7 (0.57-4.91)	0.34
International (n=26)	25	(96.2)	1 (4.0)	---	0.04
Type of drinking water					
Municipal, bottled (n=117)	96	(81.4)	21 (80.7)	1.0	
Any well water (n=27)	22	(18.6)	5 (19.2)	---	1.0
Poultry consumption					
No (n=16)	15	(14.6)	1 (3.9)	1.0	
Yes (n=113)	88	(85.4)	25 (96.2)	---	0.19

Any animal contact					
No (n=53)	47	(39.2)	6 (21.4)	1.0	
Yes (n=95)	73	(60.8)	22 (78.6)	0.4 (0.16-1.12)	0.08
Contact with livestock					
No (n=135)	107	(89.2)	28 (100.0)	1.0	
Yes (n=13)	13	(10.8)	0 (0.0)	---	0.13
Cattle density in resident county [e]					
Low <8400 cattle (n=23)	17	(19.1)	6 (40.0)	1.0	
High ≥8400 cattle (n=81)	72	(80.9)	9 (60.0)	2.8 (0.88-9.01)	0.07
Residence type					
Urban (n=117)	90	(58.4)	27 (77.1)	1.0	
Rural (n=72)	64	(41.6)	8 (22.9)	2.4 (1.02-5.62)	0.03

[a] Not all numbers add up to the total number of cases per category due to missing data for some variables or the exclusion of susceptible isolates.

[b] The 95% confidence interval (CI) for the odds ratio (OR) is presented; ORs were calculated for Cluster I relative to Clusters II and III combined.

[c] The Fisher's Exact Test was used for variables with ≤5 in one cell; no ORs were calculated

[d] Self-reported race categories in the online Michigan Disease Surveillance System questionnaire were: Caucasian, African American, Asian, American Indian/Alaska Native, Hawaiian/Pacific Islander, Unknown, or Other.

[e] Cattle density was not known for multiple counties with high case counts.
